# Fe(III)-Shikonin supramolecular nanomedicines as immunogenic cell death stimulants and multifunctional immunoadjuvants for tumor vaccination

**DOI:** 10.7150/thno.81650

**Published:** 2023-09-25

**Authors:** Wenjie Feng, Wanrui Shi, Yanqi Cui, Jiajun Xu, Shuwei Liu, Hang Gao, Shoujun Zhu, Yi Liu, Hao Zhang

**Affiliations:** 1 Joint Laboratory of Opto-Functional Theranostics in Medicine and Chemistry, The First Hospital of Jilin University, Changchun 130021, P. R. China.; 2 State Key Laboratory of Supramolecular Structure and Materials, College of Chemistry, Jilin University, Changchun 130012, P. R. China.; 3 Green Catalysis Center, College of Chemistry, Zhengzhou University, Zhengzhou 450001, P. R. China.

**Keywords:** shikonin, metal-phenolic networks, nanomedicines, immunogenic cell death, immunoadjuvants, tumor nanovaccines

## Abstract

Immunoadjuvants, as an indispensable component of tumor vaccines, can observably enhance the magnitude, breadth, and durability of antitumor immunity. However, current immunoadjuvants suffer from different issues such as weak immunogenicity, inadequate cellular internalization, poor circulation time, and mono-functional bioactivity.

**Methods:** Herein, we construct Fe^3+^-Shikonin metal-phenolic networks (FeShik) nanomedicines as immunogenic cell death (ICD) stimulants and multifunctional immunoadjuvants for tumor vaccination. The multifunctionality of FeShik nanomedicines is investigated by loading ovalbumin (OVA) as the model antigen to construct OVA@FeShik nanovaccines or 4T1 tumor cell fragment (TF) as homologous antigen to construct TF@FeShik nanovaccines. In vitro examinations including GSH responsive, •OH generation, colloid stability, cellular uptake, cytotoxicity mechanism of ferroptosis and necroptosis, ICD effect, the promotion of DC maturation and antigen cross-presentation were studied. In vivo observations including pharmacokinetics and biodistribution, antitumor effect, abscopal effect, immune memory effect, and biosafety were performed.

**Results:** The presence of FeShik nanomedicines can significantly prolong the blood circulation time of antigens, increasing the bioavailability of antigens. Upon phagocytosis by tumor cells, FeShik nanomedicines can disassemble into Fe^2+^ and Shikonin in response to tumor microenvironments, leading to ICD of tumor cells via ferroptosis and necroptosis. Consequently, ICD-released autologous tumor cell lysates and pro-inflammatory cytokines not only stimulate DC maturation and antigen cross-presentation, but also promote macrophage repolarization and cytotoxic T lymphocyte infiltration, resulting in the activation of adaptive immune responses toward solid tumors.

**Conclusion:** In a word, our FeShik supramolecular nanomedicines integrate bioactivities of ICD stimulants and immunoadjuvants, such as eradicating tumor cells, activating antitumor immune responses, modulating immunosuppressive tumor microenvironments, and biodegradation after immunotherapy. Encouraged by the diversity of polyphenols and metal ions, our research may provide a valuable paradigm to establish a large library for tumor vaccination.

## Introduction

Tumor vaccines are potential and promising immunotherapeutic strategies to activate immune system to recognize and eradicate tumor cells, meanwhile, prevent tumor recurrence and metastasis by establishing a long-term antitumor immunologic memory 1-6. Pathologically, tumor vaccines deliver antigens and immunoadjuvants to antigen-presenting cells to elicit antigen-specific adaptive immune responses 7, 8. As an indispensable component of tumor vaccines, immunoadjuvants can observably enhance the magnitude, breadth, and durability of antitumor immunity 9, 10. Despite various inorganics and organics have been applied as immunoadjuvants for tumor vaccine-based immunotherapies, these immunoadjuvants suffer from different issues. For example, most of inorganic immunoadjuvants can only stimulate T helper type-2 (Th2)-biased humoral immunity instead of Th1-biased cellular immunity because of their weak immunogenicity and inadequate cellular internalization 10-15. Organic immunoadjuvants such as toll-like receptor (TLR) agonists encounter with the short half-life experienced poor circulation time due to the rapid renal clearance and endogenous enzymatic degradation 16-18. More importantly, the single function of current immunoadjuvants cannot simultaneously complete complicated missions for antitumor immunotherapy, which include eradicating primary tumors, activating antitumor immune responses, modulating immunosuppressive tumor microenvironments, and biodegradation after immunotherapy. Thus, it is urgent to design and construct novel immunoadjuvants with a variety of biological activities for tumor vaccination cascade.

Recently, tumor vaccination depended on immunogenic cell death (ICD) is preliminarily pursued to stimulate comprehensive antitumor immune response 19, 20. ICD is a death modality accompanied by the release of autologous tumor cell lysates and pro-inflammatory cytokines, which not only stimulate and diversify tumor-specific T lymphocytes, but also reverse immunosuppressive tumor microenvironments for T lymphocyte infiltration and tumor cell eradication 21-25. Thus, evoking ICD of tumor cells is significantly beneficial to amplify the specific antitumor immunity of tumor vaccines. However, most present ICD is linked to immune-tolerogenic apoptosis, in which apoptotic cells generally maintain immune silencing 26, 27. Unlike apoptosis, ferroptosis and necroptosis are two kinds of programmed cell deaths with higher immunogenicity. Ferroptosis is characterized by the iron-mediated accumulation of lipid peroxidation (LPO) and reactive oxygen species (ROS), giving rise to the evocation of ICD by releasing DAMPs 28-33. In addition, necroptosis is mediated by receptor-interacting protein kinase-1 (RIP1), RIP3, and its substrate executioner protein mixed lineage kinase domain-like (MLKL), resulting in the membrane permeabilization and the release of intracellular contents, especially immunogenic DAMPs, to elicit ICD of tumor cells 34, 35. Thus, exploring immunoadjuvants with the capability of eliciting tumor cell ICD undergoing immunogenic necroptosis and ferroptosis can largely amplify antitumor immunity.

As one kind of widely existing natural product, polyphenols have great potential in the field of tumor therapy 36, 37. Metal ions, such as Fe^3+^, Cu^2+^, Mn^2+^, also exhibit pivotal antitumor properties upon multi-step biocatalytic reactions 38-45. Benefiting from the coordination interactions between multivalent metal ions and polyphenols, metal-phenolic networks have been speedily explored over the past decade 46-49. Unlike nanomedicines made up of covalent bonds, metal-phenolic networks not only perfectly inherit bioactivities of metal ions and polyphenols, but also attach many additional advantages such as easy preparation, huge diversity from plentiful types of polyphenols and metal ions, controlled responsiveness, and biodegradable, making metal-phenolic networks as promising nanomedicines for tumor theranostics 50, 51. Despite many metal-phenolic networks have been constructed for chemotherapy, chemodynamic therapy, and photothermal therapy of tumors, the utilization of metal-phenolic networks as ICD inducers for tumor immunotherapy is still in its infancy stage 52, 53. For example, Mn^2+^/Fe^3+^-tannic acid metal-phenolic networks are employed to elicit ICD of tumor cells 52. V-tannic acid metal-phenolic networks also serve as the photothermal agent to inhibit tumor metastasis and recurrence 54. Most recently, Dai explores several artificial polyphenol derivative-based metal-phenolic networks as ICD inducers for immunotherapy of tumors 24, 55, 56. However, most of aforementioned metal-phenolic networks overlook the bioactivities of polyphenols, but use metal-phenolic networks as the responsive nanocarriers to deliver and release metal ions. What is more, employing metal-phenolic networks as immunoadjuvants to elicit ferroptosis and necroptosis of tumor cells with stronger immunogenicity to further amplify ICD effect for antitumor immunity has still not been reported.

**Scheme 1:**
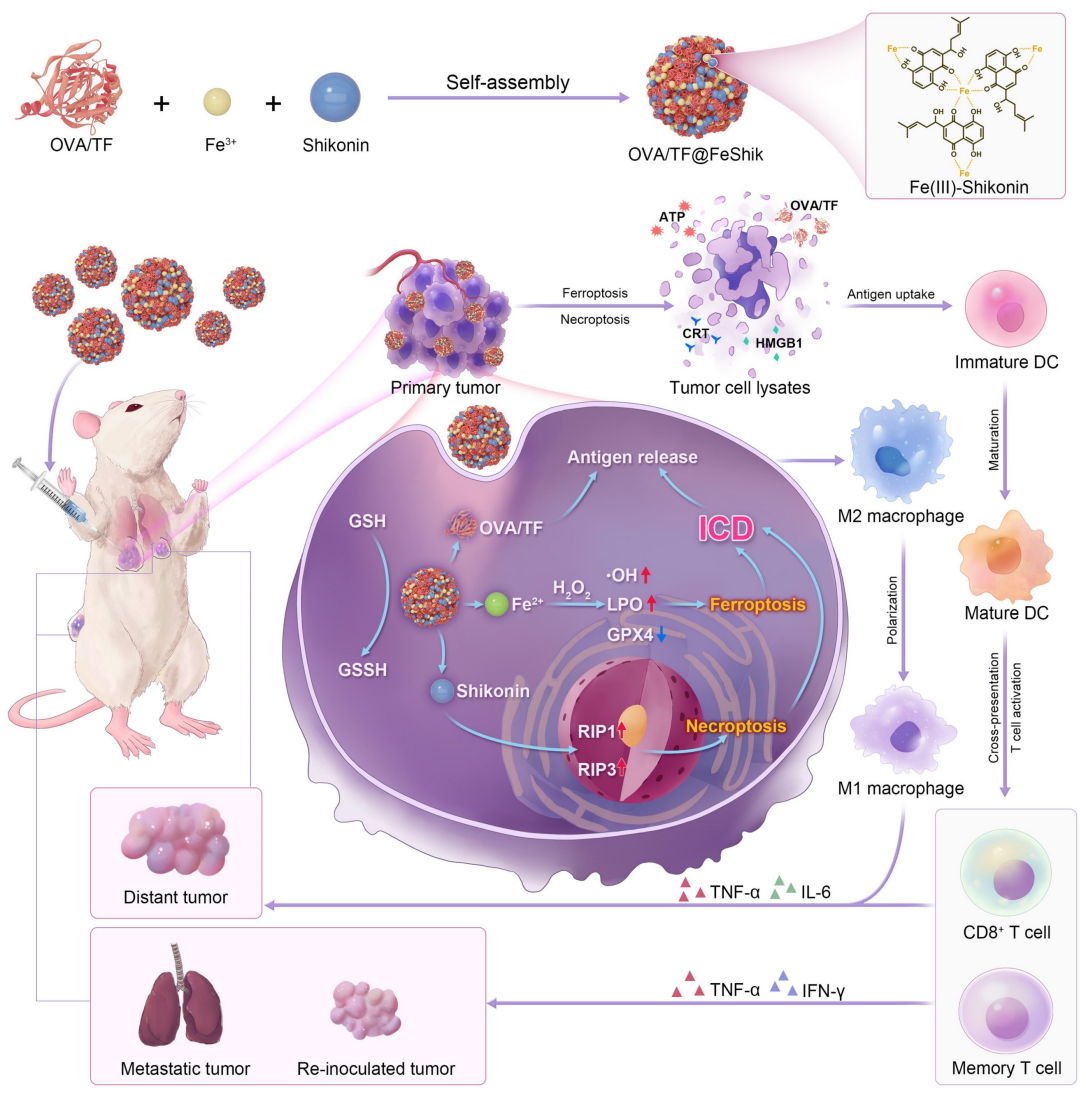
Schematic illustration of FeShik nanomedicines for tumor immunotherapy.

Herein, we construct Fe^3+^-Shikonin metal-phenolic networks (FeShik) nanomedicines as ICD stimulants and multifunctional immunoadjuvants for tumor vaccination **(Scheme 1)**. The as-prepared FeShik not only integrates the theranostics functions of Fe ions and Shikonin, but also overcomes the shortcomings of Shikonin including its low bioavailability and high toxicity toward normal tissues. Furthermore, the presence of FeShik nanomedicines can significantly prolong the blood circulation time of antigens, guaranteeing their accumulation in tumor tissues. Upon phagocytosis by tumor cells, FeShik nanomedicines will disassemble into Fe^2+^ and Shikonin in response to tumor microenvironments, leading to ICD of tumor cells via ferroptosis and necroptosis. Moreover, ICD-released autologous tumor cell lysates and pro-inflammatory cytokines not only stimulate DC maturation and antigen cross-presentation, but also promote macrophage repolarization and cytotoxic T lymphocyte infiltration, resulting in the activation of adaptive immune responses toward solid tumors. In a word, our FeShik nanomedicines fully utilize the supramolecular interactions and biological activities of Fe^3+^/Fe^2+^ and Shikonin, allowing them to perform multiple functions of ICD stimulants and immunoadjuvants, such as tumor tissue accumulation and tumor cell eradication, antitumor cellular immunity activation, modulation of immunosuppressive tumor microenvironments, as well as biodegradation after immunotherapy. Benefiting from the advantages of FeShik nanomedicines, nanovaccines prepared upon loading tumor cell fragment (TF) (TF@FeShik) exhibit an efficient antitumor effect to eradicate primary tumor, a strong abscopal effect to inhibit distance tumor growth, and a long-term immune memory effect against tumor metastasis and reoccurrence.

## Result and Discussion

### Preparation and characterization

**Figure 1:**
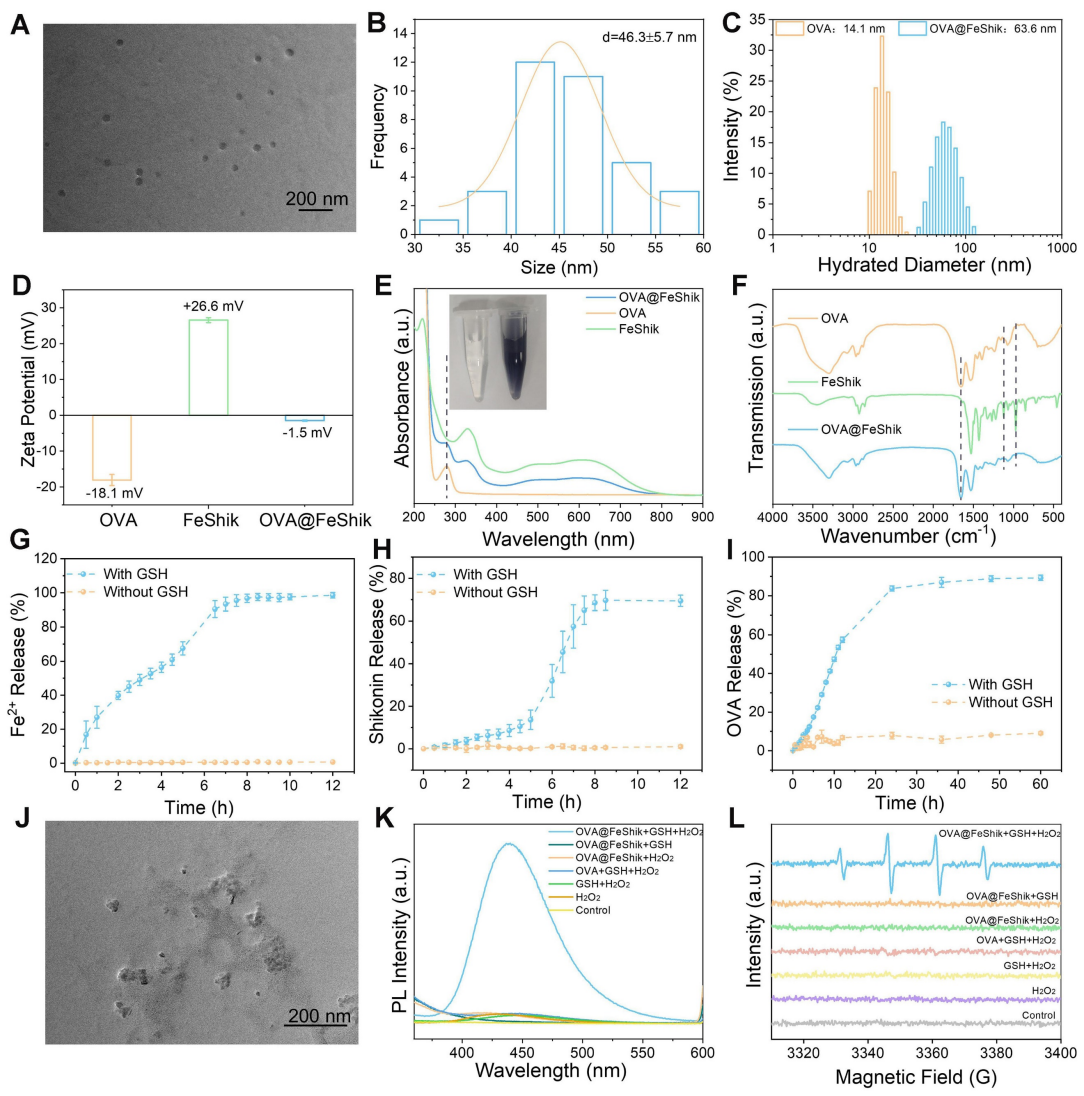
TEM image (A) and size distribution (B) of OVA@FeShik. (C) Hydrated diameter of OVA and OVA@FeShik. (D) Zeta potential statistics of OVA, FeShik, and OVA@FeShik. (E) UV-vis absorption spectra of OVA@FeShik, OVA, and FeShik. Inset in (E): photographs of OVA and OVA@FeShik solution. (F) FTIR spectra of OVA, FeShik, and OVA@FeShik. Accumulative Fe^2+^ (G), Shikonin (H), and OVA (I) release with or without GSH (n = 3). (J) TEM image of OVA@FeShik after treating with GSH. (K) Fluorescence spectra of TPA to detect the generation of •OH under different treatments. (L) ESR spectra of DMPO to detect the generation of •OH under different treatments. Data are shown as mean ± SD; n represents the number of independent samples.

We first investigate the multifunctionality of FeShik nanomedicines by loading OVA as the model antigen via the coordination interactions between Fe^3+^ and amino acid in OVA, as well as the strong binding affinity of carbonyl and phenolic groups in Shikonin toward proteins (OVA@FeShik nanovaccines). The as-prepared OVA@FeShik nanovaccines are monodispersed nanospheres with an average diameter of 46.3 ± 5.7 nm (**Figure 1A-B**). The hydrated diameter of OVA@FeShik nanovaccines is 63.6 nm, which is significantly larger than that of FeShik or pure OVA (**Figure 1C** and **S1**). Since the zeta potentials of FeShik and OVA are +26.6 and -18.1 mV, respectively, the zeta potential of OVA decreases to -1.50 mV after conjugating with FeShik nanomedicines (**Figure 1D**). **Figure 1E** shows the ultraviolet-visible (UV-vis) absorption spectrum of OVA@FeShik nanovaccines. The appearances of characteristic absorption peaks at 269 and 450~800 nm attribute to OVA and FeShik, suggesting that OVA@FeShik nanovaccines are composed of OVA and FeShik. Fourier transform infrared (FTIR) spectra exhibit the emergence of the vibration peaks belonging to FeShik (1120 and 971 cm^-1^) and the stretching vibration peak of C=O belonging to OVA (1650 cm-1), further confirming the presence of OVA and FeShik in OVA@FeShik nanovaccines (**Figure 1F**). Based on the standard curve in **Figure S2**, the loading efficiency and loading content of OVA in OVA@FeShik nanovaccines calculate to be as high as 33.9 wt% and 72.3 wt%. The loading content is much higher than other tumor nanovaccines 19, 57.

Because Fe^3+^ will be reduced into Fe^2+^ upon the exposure of a high concentration of GSH, followed by the destruction of the coordination between Fe^3+^ and Shikonin. **Figure 1G**-**I** and** S2**-**S4** show the release profiles of Fe^2+^, Shikonin, and OVA from OVA@FeShik nanovaccines after incubation with 10 mM GSH. As a result, all of them continually release as a function of the incubation time. Since Fe^2+^ has a smaller size than Shikonin and OVA, it has the fastest release rate during the disassembly of OVA@FeShik nanovaccines. In sharp contrast, nearly no Fe^2+^, Shikonin, or OVA is released from OVA@FeShik nanovaccines in the absence of GSH. The transmission electron microscopy (TEM) image in **Figure 1J** depicts the disassembly of OVA@FeShik nanovaccines in the presence of GSH as well. Because Fe^2+^ can catalyze H_2_O_2_ into •OH via Fenton reaction, disodium terephthalate is used as the probe to inspect the generation of •OH. Fluorescence belonging to 2-hydroxyterephthalic disodium (an oxidation product of disodium terephthalate) can only be detected in aqueous solution containing OVA@FeShik, GSH, and H_2_O_2_ simultaneously (**Figure 1K**). Electron spin resonance (ESR) is employed to inspect •OH by using 5,5-dimethyl-1-pyrroline N-oxide (DMPO) as the trapping agent (**Figure 1L**). The typical 1:2:2:1 characteristic signal of •OH can only be observed in the presence of OVA@FeShik, GSH, and H_2_O_2_ simultaneously. All these results demonstrate that FeShik nanomedicines can prevent the leakage of antigens from nanovaccines in normal physiological environments, but specifically degrade to release antigens after exposure to the tumor microenvironments, resulting in the depletion of GSH and the release of Fe^2+^ to promote the generation of •OH via Fenton reaction.

### Colloid stability and cytotoxicity

The colloidal stabilities of OVA@FeShik nanovaccines at multiple physiological environments are investigated. **Figure S5** shows OVA@FeShik nanovaccines restored in water, PBS, and 1640 basic for 7 days, which all possess stable hydrated diameters without obvious aggregation. Subsequently, the cytotoxicity of OVA@FeShik nanovaccines toward 4T1 murine breast cancer cells (tumor cells) and L929 mouse fibroblast cells (normal cells) are studied via cell counting kit-8 (CCK-8) assay. The viability of 4T1 cells declines rapidly with increasing the concentration of OVA@FeShik nanovaccines. Only 31.8% of 4T1 cells survive after incubation with 75 μg/mL of OVA@FeShik nanovaccines (**Figure S6A**). In contrast, the viability of L929 cells is as high as 96.9% under the same condition (**Figure S6B**). **Figure S6C**-**D** shows the GSH levels in 4T1 and L929 cells under the OVA@FeShik treatment. The content of GSH in 4T1 cells decreases with increasing the concentration of OVA@FeShik. When the concentration of OVA@FeShik reaches 75 μg/mL, the GSH content decreases to 34.9%. However, the GSH level in L929 cells is above 80% under the same treatment. Because free OVA have no significant cytotoxicity on both of L929 and 4T1 cells (**Figure S6E**-**F**), the selective cytotoxicity of OVA@FeShik nanovaccines mainly comes from FeShik nanomedicines, which disassemble into Fe^2+^ and Shikonin upon the exposure of the high intracellular GSH level in 4T1 cells.

Localization analysis is conducted to investigate cellular uptake, intracellular distribution, and endosomal escape capability of OVA@FeShik nanovaccines in 4T1 cells. **Figure S7A** shows confocal laser scanning microscopy (CLSM) images of 4T1 cells after incubation with fluorescein isothiocyanate (FITC)-labeled OVA (^FITC^OVA) or OVA@FeShik constructed by FITCOVA (^FITC^OVA@FeShik). Unlike FITCOVA, ^FITC^OVA@FeShik prefer to be internalized by 4T1 cells. The cellular uptake efficiency of ^FITC^OVA@FeShik is 2.4-fold higher than that of ^FITC^OVA after incubation for 6 h (**Figure S7B**). More importantly, ^FITC^OVA@FeShik exhibits a distinct endo/lysosomal escape capability. Generally, a Pearson's correlation coefficient R-value of 0.93 indicates that most of the internalized FITCOVA are colocalized with endo/lysosomal, represented by the orange fluorescence (overlap of red and green fluorescence) in the merged CLSM images. However, the cells display segregated green fluorescence after incubation with ^FITC^OVA@FeShik, suggesting the successful endo/lysosomal escape of ^FITC^OVA@FeShik, and the Pearson's R-value reduces to 0.57 (**Figure S7C-D**). Thus, it is suggested that FeShik nanomedicines can promote effective tumor cell uptake and subsequent endo/lysosomal escape of antigens, which is better than using OVA and FeShik in combination for executing their immunotherapy performances.

The cytotoxicity mechanism of OVA@FeShik nanovaccines toward 4T1 cells is investigated. According to our previous report, FeShik can release Fe^2+^ and Shikonin to produce ROS under the exposure of GSH 58, **Figure S8** shows intracellular ROS levels by using 2',7'-dichlorofluorescin diacetate (DCFH-DA) as the fluorescence probe. No obvious green fluorescence can be seen in 4T1 cells treated by OVA. 4T1 cells under FeShik or OVA@FeShik treatment show bright green fluorescence, indicating the ability of FeShik nanomedicines on the generation of ROS. Intracellular ROS level after FeShik or OVA@FeShik treatment is further quantified, which is 5.1-fold and 5.0-fold higher than that in Control, respectively. **Figure S9** shows the viability of 4T1 cells in the presence of different inhibitors after incubation with OVA@FeShik. Instead of APO (apoptosis inhibitor), Fer-1 (ferroptosis inhibitor) and Nec-1 (necroptosis inhibitor) can effectively prevent 4T1 cells from death, suggesting that ferroptosis and necroptosis are dominant cell death pathways elicited by OVA@FeShik. Ferroptosis of 4T1 cells is further confirmed by monitoring intracellular Fe^2+^, GSH, GPX4, and LPO levels. Owing to the redox reaction between Fe^3+^ and GSH, FeShik and OVA@FeShik can significantly elevate Fe^2+^ level in 4T1 cells but dramatically consume intracellular GSH (**Figure S10** and** S11**). In the meantime, the depletion of GSH will down-regulate the expression level of GPX4, consistent with the results of immunofluorescence analysis, enzyme-linked immunosorbent assay (ELISA) test, and western blot measurement (**Figure S12**). Because GPX4 is vital in removing lethal lipid peroxides (LPO), BODIPY^581/591^-C11 staining assay shows that FeShik and OVA@FeShik can boost LPO accumulation in 4T1 cells (**Figure S13**). All these analyses demonstrate that FeShik nanomedicines can induce 4T1 cell death via ferroptosis.

Because the release of Shikonin from FeShik can elicit necroptosis, the expression levels of necroptosis-related proteins, such as RIP1 and RIP3 are analyzed to evaluate 4T1 cell necroptosis under different treatments. Immunofluorescence analysis, ELISA test, and western blot measurement all reveal the up-regulation of RIP1 and RIP3 in 4T1 cells after FeShik or OVA@FeShik treatment (**Figure S14**-**S17**). Flow cytometry also shows an OVA@FeShik concentration-dependent necroptosis enhancement (**Figure S18**). These results demonstrate that FeShik nanomedicines can elicit 4T1 cell necroptosis.

### ICD effect

**Figure 2:**
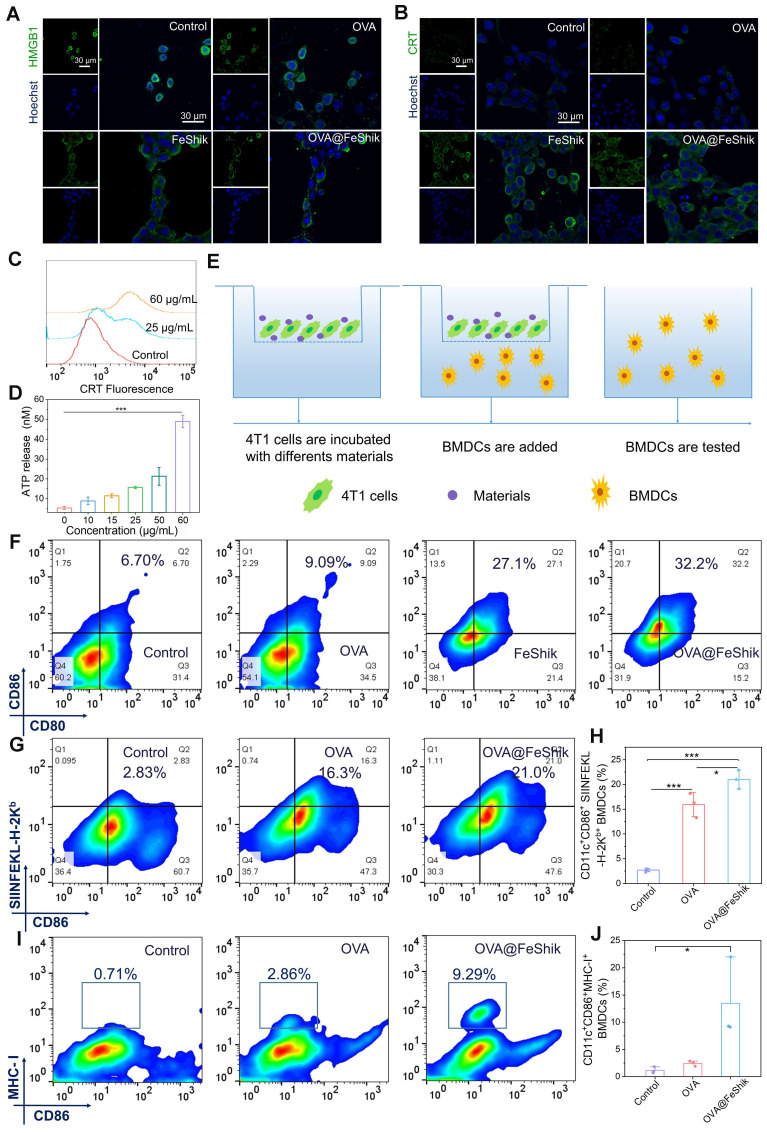
CLSM images of HMGB1 translocation (A) and CRT exposure (B) in 4T1 cells after different treatments. (C) Flow cytometric analysis of CRT exposure in 4T1 cells after treating by different concentrations of OVA@FeShik. (D) ATP secretion levels of 4T1 cells after treating with different concentrations of OVA@FeShik (n = 3). (E) Schematic illustration of the coculture system of 4T1 cell residues and immature DC in vitro. (F) Flow cytometric analysis of DC maturation after different treatments. Flow cytometric analysis (G) and corresponding quantification (H) of CD86^+^ SIINFEKL-H-2K^b+^ DCs (gated on CD11c^+^) after different treatments (n = 3). Flow cytometric analysis (I) and corresponding quantification (J) of CD86^+^MHC-I^+^ DCs (gated on CD11c^+^) after different treatments (n = 3). Data are shown as mean ± SD; n represents the number of biologically independent samples. *p < 0.05, **p < 0.01, and ***p < 0.001.

Ferroptosis and necroptosis can trigger ICD of tumor cells. Thus, ICD-related distinct biochemical hallmarks including high-mobility group protein B1 (HMGB1), calreticulin (CRT), and adenosine triphosphate (ATP) are analyzed to evaluate the ICD effect of OVA@FeShik nanovaccines 59. Unlike untreated or OVA-treated 4T1 cells, whose HMGB1 mainly locate in nucleus, HMGB1 in FeShik- or OVA@FeShik-treated 4T1 cells leak from nucleus and translocate outside nucleus, suggesting the ICD inducibility of FeShik nanomedicines (**Figure 2A**). Moreover, the bright green immunofluorescence of CRT appears on membranes of 4T1 cells treated by FeShik or OVA@FeShik (**Figure 2B**). In contrast, free OVA have no effect on the exposure of CRT, which further identify that FeShik nanomedicines can elicit ICD of 4T1 cells. The CRT exposure level induced by OVA@FeShik significantly increases with increasing their concentration (**Figure 2C**). Upon increasing the concentration of OVA@FeShik to 25 and 60 µg/mL, the corresponding CRT exposure levels are 2.7-fold and 4.8-fold higher than cells in Control. Western blot analysis also shows that cytoplasmic HMGB1 and CRT are significantly enhanced in FeShik and OVA@FeShik groups (**Figure S19**). Subsequent detection of ATP secretion exhibits the same variation tendency. OVA@FeShik can remarkably increase ATP secretion levels in a concentration-dependent manner. ATP secretion in cells treated by 60 µg/mL of OVA@FeShik is 9.2-fold higher than that in 4T1 cells in Control (**Figure 2D**). All these results firmly demonstrate that FeShik nanomedicines can efficiently trigger ICD of 4T1 cells via ferroptosis and necroptosis.

ICD-released autologous tumor cell lysates can be engulfed by immature DCs to stimulate their maturation. We establish a Transwell system to study DC maturation induced by OVA@FeShik nanovaccines (**Figure 2E**). 4T1 tumor cells in the upper chamber are subjected to various treatments: (i) Control, (ii) OVA, (iii) FeShik, (iv) OVA@FeShik. Afterward, bone-marrow-derived dendritic cells (BMDCs) are collected and seeded in the lower chamber and co-incubated with 4T1 cells. The expression of the mature marker (CD11c^+^CD80^+^CD86^+^) on BMDCs is determined by flow cytometry. Compared to cells in Control, only a slight increase can be observed in cells treated by OVA owing to its immunogenicity (**Figure 2F**). However, the percentage of matured DCs increases to 27.1% after FeShik treatment, which is 4.0-fold higher than that in Control. This result indicates that FeShik-caused ICD can strongly stimulate DC maturation. Surprisingly, OVA@FeShik can further elevate the percentage of matured DCs to 32.2%, demonstrating the synergetic effect between antigens and immunoadjuvants.

In addition, the antigen presentation process from nanovaccines to antigen-presenting cells is inspected with the help of ^FITC^OVA@FeShik. **Figure S20** shows the CLSM images of BMDCs and bone-marrow-derived macrophages (BMDMs) in the lower chamber of the Transwell system. The bright green fluorescence in BMDCs and BMDMs demonstrate that ^FITC^OVA loaded by FeShik nanomedicines can be successfully released from dying tumor cells and phagocytized by antigen-presenting cells, which is beneficial for activating T lymphocytes for antigen-specific immune responses against solid tumors. Then, we use the fluorescent-tagged anti- SIINFEKL-H-2K^b^ antibody to specifically bind to major histocompatibility complex class I molecules (MHC-I) presenting a fragment of OVA 60. As a result, the ratio of CD11c^+^CD86^+^ SIINFEKL-H-2K^b+^ BMDCs in OVA@FeShik group is 1.3 times higher than that in OVA group (**Figure 2G**-**H**). Moreover, the ratio of CD11c^+^CD86^+^MHC-I^+^ BMDCs in OVA@FeShik group is 5.5 times higher than that in OVA group (**Figure 2I**-**J**), further confirming the immunoadjuvant capability of FeShik nanomedicines on stimulating antigen cross-presentation through MHC-I-mediated pathway. Taken together, FeShik nanomedicines can not only deliver antigens, but also elicit ICD of 4T1 cells, leading to the release of autologous tumor cell lysates, and the promotion of DC maturation and antigen cross-presentation.

### Pharmacokinetics and biodistribution

**Figure 3:**
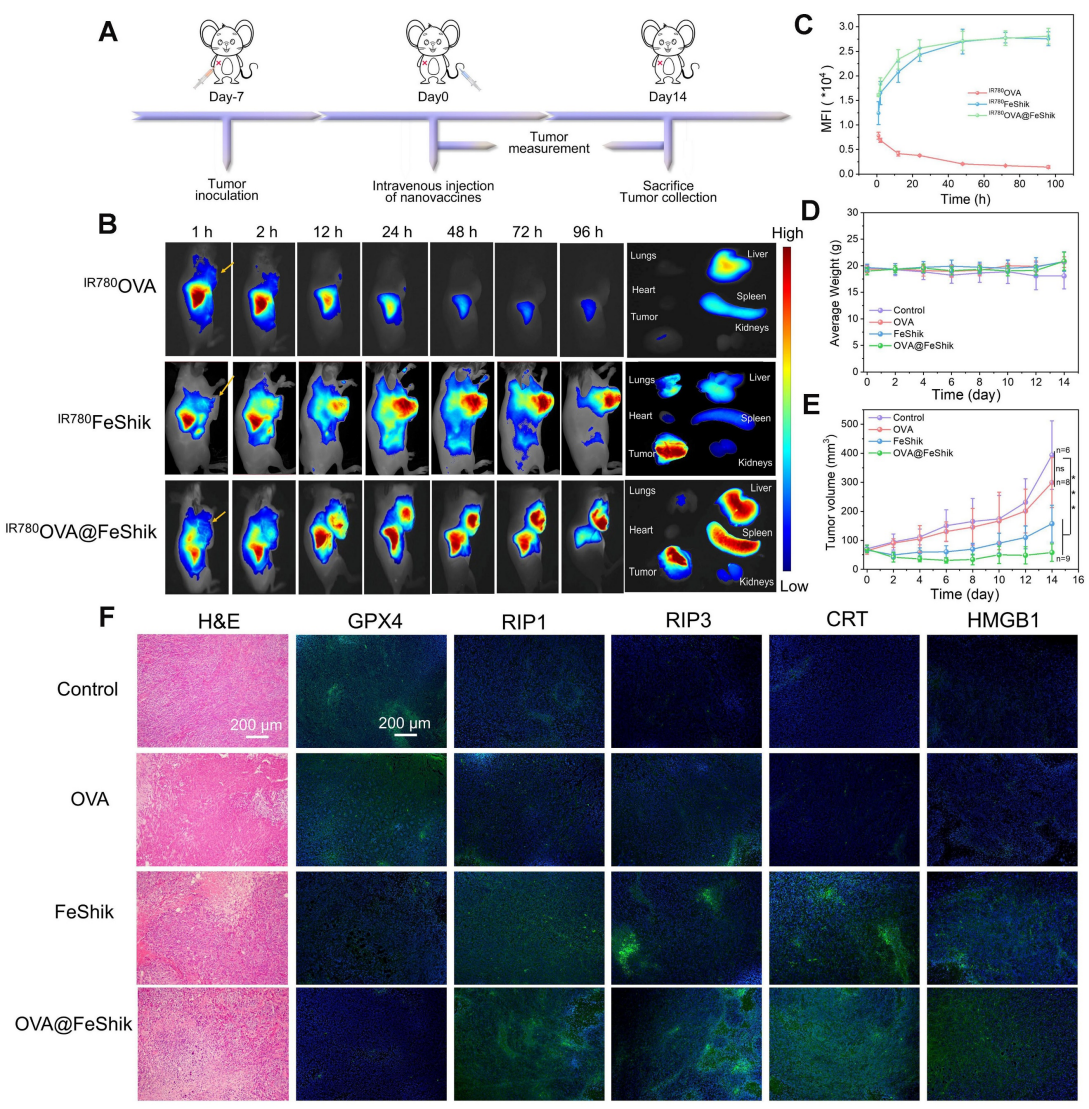
(A) Schematic illustration of the treatment protocol in orthotopic 4T1 tumor models. (B) In vivo fluorescence images of orthotopic 4T1 tumor-bearing mice after intravenous injection of ^IR780^OVA,^ IR780^FeShik, or^ IR780^OVA@FeShik for different times (yellow arrows pointing tumors) and ex vivo fluorescence image of tumor and major organs dissected from mice after intravenous injection for 96 h. (C) Accumulation curves of ^IR780^OVA, ^IR780^FeShik, and^ IR780^OVA@FeShik in tumor tissues by measuring the fluorescence intensity of tumors at different time points (n = 3). (D) Average body weight of mice with various treatments. (E) Average tumor growth curves of 4T1 tumors on the orthotopic 4T1 tumor-bearing mice with various treatments. (F) H&E staining and immunofluorescence staining (GPX4, RIP1, RIP3, CRT and HMGB1) images of tumor tissue slices from mice sacrificed at 14th day after different treatments. Data are shown as mean ± SD; n represents the number of biologically independent samples. *p < 0.05, **p < 0.01, and ***p < 0.001.

Pharmacokinetics and biodistribution profiles of OVA@FeShik nanovaccines are evaluated after intravenous injection into orthotopic 4T1 tumor-bearing female BALB/c mice. IR780-labeled OVA (^IR780^OVA) is used to construct ^IR780^OVA@FeShik nanovaccines with a near-infrared (NIR) fluorescence. By recording the IR780 fluorescence of blood samples withdrawn from mice at varying time intervals post-intravenous injection, the half-life times (*t*_½_) of^ IR780^OVA, ^IR780^FeShik, and ^IR780^OVA@FeShik are calculated to be 30.9 ± 3.1, 200.8 ± 44.3, and 182.0 ± 24.2 min, respectively (**Figure S21**). The greatly increased blood circulation time of ^IR780^OVA@FeShik compared to ^IR780^OVA is mostly due to the superior colloidal stability of FeShik nanomedicines, which ensures the simultaneous accumulation of antigens in tumor tissues via EPR effect.** Figure 3B**-**C** shows in vivo fluorescence images of mice after intravenous injection of ^IR780^OVA, ^IR780^FeShik, or ^IR780^OVA@FeShik. Like ^IR780^FeShik,^ IR780^OVA@FeShik are gradually accumulated in tumor tissues during the initial 12 h, peaked at 48th h post-injection, and maintained at a high level even after injection for 96 h. In stark contrast,^ IR780^OVA-injected mice exhibit negligible fluorescence signal in tumor tissues during the entire monitoring period. Most of ^IR780^OVA concentrate in liver, followed by rapid metabolism after intravenous injection for 24 h due to their relatively small size. Ex vivo fluorescence images of main organs and tumors are dissected and harvested at 96th h post-injection for semi-quantitative analysis. The fluorescence intensity of tumors treated by ^IR780^FeShik or ^IR780^OVA@FeShik is 12.1-fold or 13.1-fold higher than that treated by ^IR780^OVA (**Figure S22**). Notably, liver and spleen in^ IR780^OVA@FeShik group exhibit a bright fluorescence as well. The accumulation of ^IR780^OVA@FeShik in liver and spleen can be attributed to two aspects. On the one hand, ^IR780^OVA@FeShik are easy to be captured by the reticuloendothelial system owing to their size. On the other hand, released ^IR780^OVA from^ IR780^OVA@FeShik may migrate to spleen to stimulate DC maturation. The analyses above illustrate that FeShik nanomedicines can significantly increase the bioavailability of antigens, conducive to augment adaptive immunity.

### Antitumor effect

In vivo antitumor efficacy of OVA@FeShik nanovaccines is assessed in 4T1 orthotopic tumor model (**Figure 3A**). When tumors reached ~50 mm^3^, mice are randomly divided into 4 groups according to different treatments: (i) Control, (ii) OVA, (iii) FeShik, (iv) OVA@FeShik. The body weights with acceptable fluctuations imply low side effects of our treatments (**Figure 3D**). **Figure 3E** records the tumor volume changes in each group. Tumors in group (i) and (ii) grow rapidly without significant difference. FeShik can clearly inhibit tumor growth with the inhibitory efficiency of 60.1%. Notably, OVA@FeShik shows the best inhibition effect for tumor growth. The tumor volume in OVA@FeShik group is only 58.0 ± 30.1 mm^3^ at 14th day, which is 6.8-fold smaller than that in Control. **Figure S23** shows photographs of treated tumors in each group, which further presents the higher antitumor activities of FeShik and OVA@FeShik than other treatments. Notably, the better antitumor effect of OVA@FeShik than FeShik may attribute to the immunogenicity of OVA, which can activate antitumor immune responses to a larger extent. Tumors at the end of treatments are collected for histopathology and immunohistochemistry analyses. **Figure 3F** shows hematoxylin-eosin (H&E)-stained tumor slice images in each group. The necrosis areas in tumor tissues reveal the following tendency: group (iv) > group (iii) > group (ii) > group (i), suggesting the superior antitumor effect of OVA@FeShik. Moreover, the expression levels of GPX4, RIP1, and RIP3 are assessed as well. The significant down-regulation of GPX4 and up-regulation of RIP1 and RIP3 in tumors treated by FeShik or OVA@FeShik indicate that FeShik can efficiently elicit ferroptosis and necroptosis of 4T1 cells. More importantly, the expression levels of CRT and HMGB1 in FeShik and OVA@FeShik groups are significantly elevated, suggesting that FeShik can effectively induce ICD via ferroptosis and necroptosis. In a word, FeShik nanomedicines can eradicate primary tumors upon ferroptosis and necroptosis-induced ICD, greatly boosting the antitumor therapeutic efficacy of OVA@FeShik nanovaccines.

**Figure 4:**
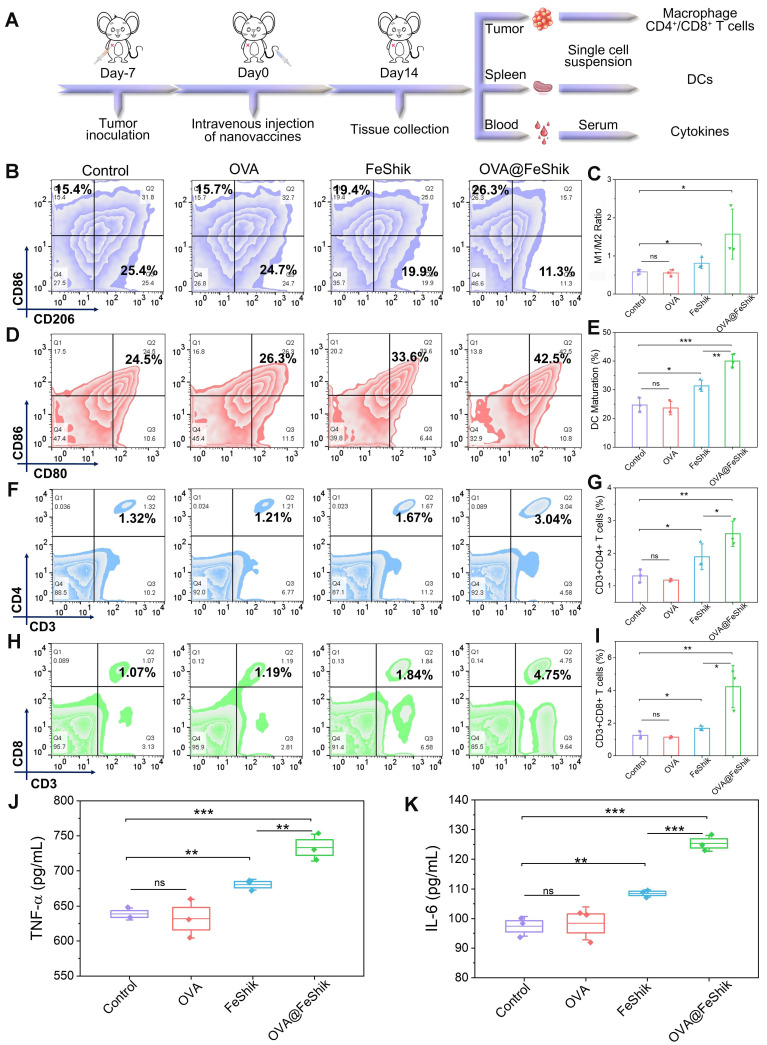
(A) Schematic illustration of the immunity analysis protocol in orthotopic 4T1 tumor models. (B) Flow cytometric analysis of the percentages of M1-phenotype macrophages (F4/80^+^CD206^-^CD86^+^) and M2-phenotype macrophages (F4/80^+^CD206^+^CD86^-^) in tumors after different treatments. (C) Statistical analysis of the ratio of M1-phenotype macrophages to M2-phenotype macrophages in tumors after different treatments (n = 3). Flow cytometric analysis (D) and corresponding quantification (E) of DC maturation (CD11c^+^CD80^+^CD86^+^) in spleens after different treatments (n = 3). Flow cytometric analysis (F) and corresponding quantification (G) of T helper cells (CD3^+^CD4^+^) in tumors after different treatments (n = 3). Flow cytometric analysis (H) and corresponding quantification (I) of cytotoxic T lymphocytes (CD3^+^CD8^+^) in tumors after different treatments (n = 3). Secretion levels of TNF-α (J) and IL-6 (K) in serum after different treatments (n = 3). Data are shown as mean ± SD; n represents the number of biologically independent samples. *p < 0.05, **p < 0.01, and ***p < 0.001.

Subsequently, single-cell suspensions from tumors and spleens are collected to evaluate the immune activation performances of OVA@FeShik nanovaccines (**Figure 4A**). Reeducating tumor-associated M2-phenotype macrophages to tumoricidal M1-phenotype macrophages denotes an effective strategy to reverse the immunosuppressive tumor microenvironments for antitumor immunity 61, 62. **Figure 4B** and** S24** show the percentages of M1- and M2-phenotype macrophages in tumor tissues under different treatments. No obvious difference can be observed between OVA group and Control. Because FeShik can cause ICD to release autologous tumor cell lysates, there is a significant increase in the relative number of M1-phenotype macrophages (F4/80^+^CD206^-^CD86^+^) from 15.1 ± 0.8% to 17.5 ± 1.3% and a substantial decrease of M2-phenotype macrophages (F4/80^+^CD206^+^CD86^-^) from 25.9 ± 0.9% to 21.8 ± 1.4% in FeShik-treated tumors. Amazingly, benefiting from the immunogenicity of OVA, the percentage of M1-phenotype macrophages in OVA@FeShik group further increases to 22.1 ± 3.1%, accompanied by the decrease of M2-phenotype macrophages to 14.9 ± 2.7%. As a result, the M1/M2 ratio in OVA@FeShik group is 1.9-fold, 2.8-fold, and 2.7-fold higher than that in FeShik, OVA, and Control groups (**Figure 4C**).

DC maturation is a crucial factor in initiating regulation and maintaining adaptive immune responses 63, 64. **Figure 4D**-**E** shows the DC maturation levels in spleens under different treatments. Similar to macrophage polarization assay, OVA alone have no influence on DC maturation. The percentage of matured DCs (CD11c^+^CD80^+^CD86^+^) increases from 24.7 ± 2.0% to 31.4 ± 1.6% under FeShik treatment owing to its strong ICD effect. Moreover, OVA@FeShik elicit the highest level of DC maturation (40.0 ± 1.9%), probably due to the synergetic effect between antigens and immunoadjuvants. The activation of DCs can stimulate cytotoxic T lymphocytes to accelerate adaptive immunity toward solid tumors 29. We then profile the lymphocytic infiltrates in tumor tissues. Flow cytometry analysis shows that FeShik can obviously up-regulate the expression frequencies of T helper cells (CD3^+^CD4^+^) (CD4^+^ T cells) and cytotoxic T lymphocytes (CD3^+^CD8^+^) (CD8^+^ T cells) in tumor tissues. And 2.60 ± 0.32% of CD4^+^ T cells and 4.22 ± 1.05% of CD8^+^ T cells are recruited into tumor tissues treated by OVA@FeShik, which is 2.0-fold and 3.4-fold higher than that in Control (**Figure 4F**-**I**). Immunofluorescence staining images further confirm the wide distribution of CD4^+^ and CD8^+^ T cells in tumor tissues treated by FeShik or OVA@FeShik (**Figure S25** and** S26)**. In addition, immune-associated proinflammatory cytokines including tumor necrosis factor α (TNF-α) and interleukin-6 (IL-6) in serum are analyzed via ELISA 65. As expected, FeShik can dramatically increase the secretion of TNF-α and IL-6, while TNF-α and IL-6 levels after OVA@FeShik treatment are the highest (**Figure 4J**-**K**). All these results demonstrate that FeShik nanomedicines can activate immune responses and reverse immunosuppressive tumor microenvironments, leading to the amplification of antitumor immunity.

### Abscopal effect

After preliminary verifying the multifunctionality of FeShik nanomedicines, 4T1 TF instead of OVA is employed as a homologous antigen to construct TF@FeShik nanovaccines 19, 57, 66. **Table S1** shows the protein composition of extracted TF characterized by UHPLC-MS/MS analysis. There are 2451 proteins in TF and the protein ID P16045 named Galectin-1 has the highest content among all the proteins. The as-prepared TF@FeShik nanovaccines are monodispersed nanospheres with an average diameter of 46.2 ± 6.0 nm and a hydration size of 62.9 nm (**Figure S27A**-**C**). The appearance of characteristic absorption peak belonging to TF (257 nm) (**Figure S27D**) and the similar protein profile to TF (10-170 kDa) via Coomassie Brilliant Blue staining (**Figure S28**) indicate the successful loading of TF in TF@FeShik nanovaccines. The corresponding loading efficiency of TF in TF@FeShik nanovaccines calculates to be as high as 65.8 wt% (**Figure S29**). Similar to OVA@FeShik, TF@FeShik possess the continual release of Fe^2+^, Shikonin, and TF in response to GSH, but no apparent release in the absence of GSH (**Figure S30**).

**Figure 5:**
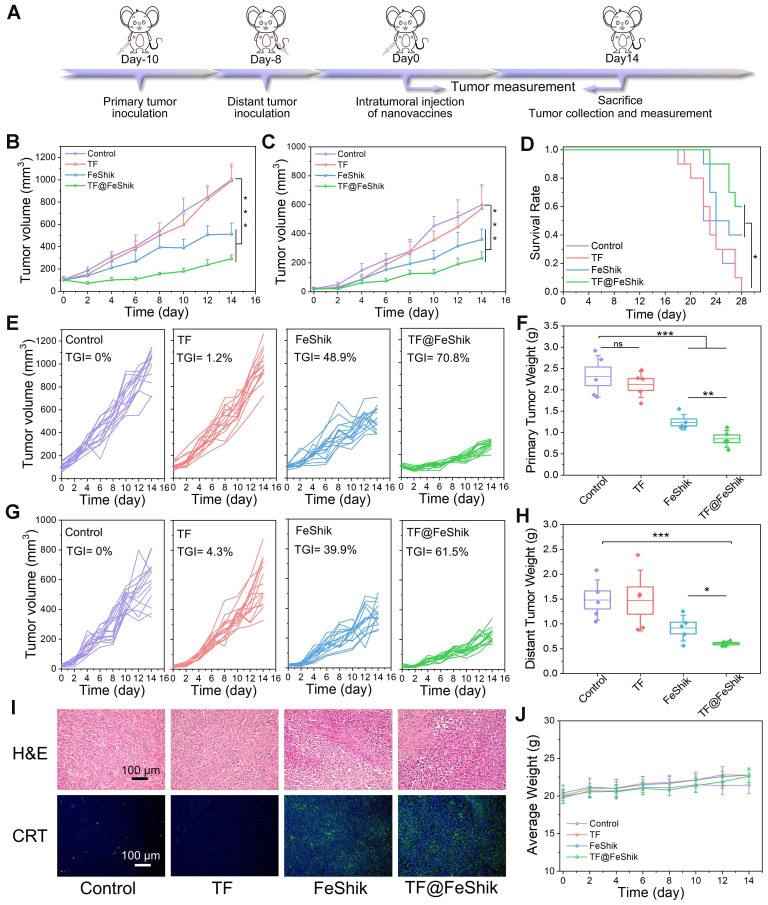
(A) Schematic illustration of the treatment protocol in bilateral 4T1 tumor model. Average tumor growth curves (B) and corresponding individual growth curves (E) of primary 4T1 tumors under various treatments (n = 15). Average tumor growth curves (C) and corresponding individual growth curves (G) of distant 4T1 tumors under various treatments (n = 15). (D) Survival curves of mice under different treatments (n = 10). Average weight of primary (F) and distant (H) tumors in each group after various treatments (n = 5). (I) H&E staining and CRT immunofluorescence staining images of primary tumor tissue slices from mice sacrificed at 14th day after different treatments. (J) Average body weight curves of mice in each group under various treatments (n = 15). Data are shown as mean ± SD; n represents the number of biologically independent samples. *p < 0.05, **p < 0.01, and ***p < 0.001.

Then, a bilateral orthotopic 4T1 tumor model is established to explore the abscopal effect of TF@FeShik nanovaccines in vivo (**Figure 5A**). The bilateral orthotopic 4T1 tumor-bearing BALB/c mice are divided into 4 groups. The primary tumors with a mean volume of ~100 mm^3^ are intratumorally injected with (ii) TF, (iii) FeShik, (iv) TF@FeShik, and (i) without any treatment as Control. The weights as well as the volumes of primary and distant tumors in each group are recorded every 2 days (**Figure 5B**-**C**,**E**,**G**). According to the growth profiles, the primary and distant tumors in Control grow rapidly, and reach ~1000 and ~600 mm^3^ on 14th day. Due to the immunosuppression tumor microenvironments and the poor immunogenicity of TF, pristine TF has no inhibitory effect on tumor growth. Because FeShik can elicit ferroptosis and necroptosis, leading to the occurrence of ICD for immune responses, tumors in FeShik group are clearly inhibited with the tumor inhibitory rate of 48.9% (primary tumor) and 39.9% (distant tumor). Benefiting from the synergetic effect between FeShik nanomedicines and TF antigens, TF@FeShik nanovaccines exhibit the highest tumor inhibitory rates on primary (70.8%) and distant (61.5%) tumors. What's more, the survival rate of mice in TF@FeShik group is remarkably improved (60%), which is significantly higher than that in other groups (**Figure 5D**). At the end of treatments, mice are euthanized to harvest all tumors for histopathology and immunohistochemistry analyses.

**Figure 5I** and** S31** show the H&E-stained slice images of primary and distant tumors. The necrosis areas in primary and distant tumors follow the same tendency: group (iv) > group (iii) > group (ii) ≈ group (i). The expression levels of GPX4, RIP1, RIP3, CRT, and HMGB1 in primary tumors are exhibited in **Figure 5I** and** S32**, consistent with the result in **Figure 3F**. **Figure S33**, **5F** and** 5H** present the photographs and weights of bilateral tumors in each group, which further prove the superior antitumor activity of TF@FeShik nanovaccines. The body weight of mice shows a gradual increase during treatments, implying no significant side effects of the treatments (**Figure 5J** and** S34**).

**Figure 6:**
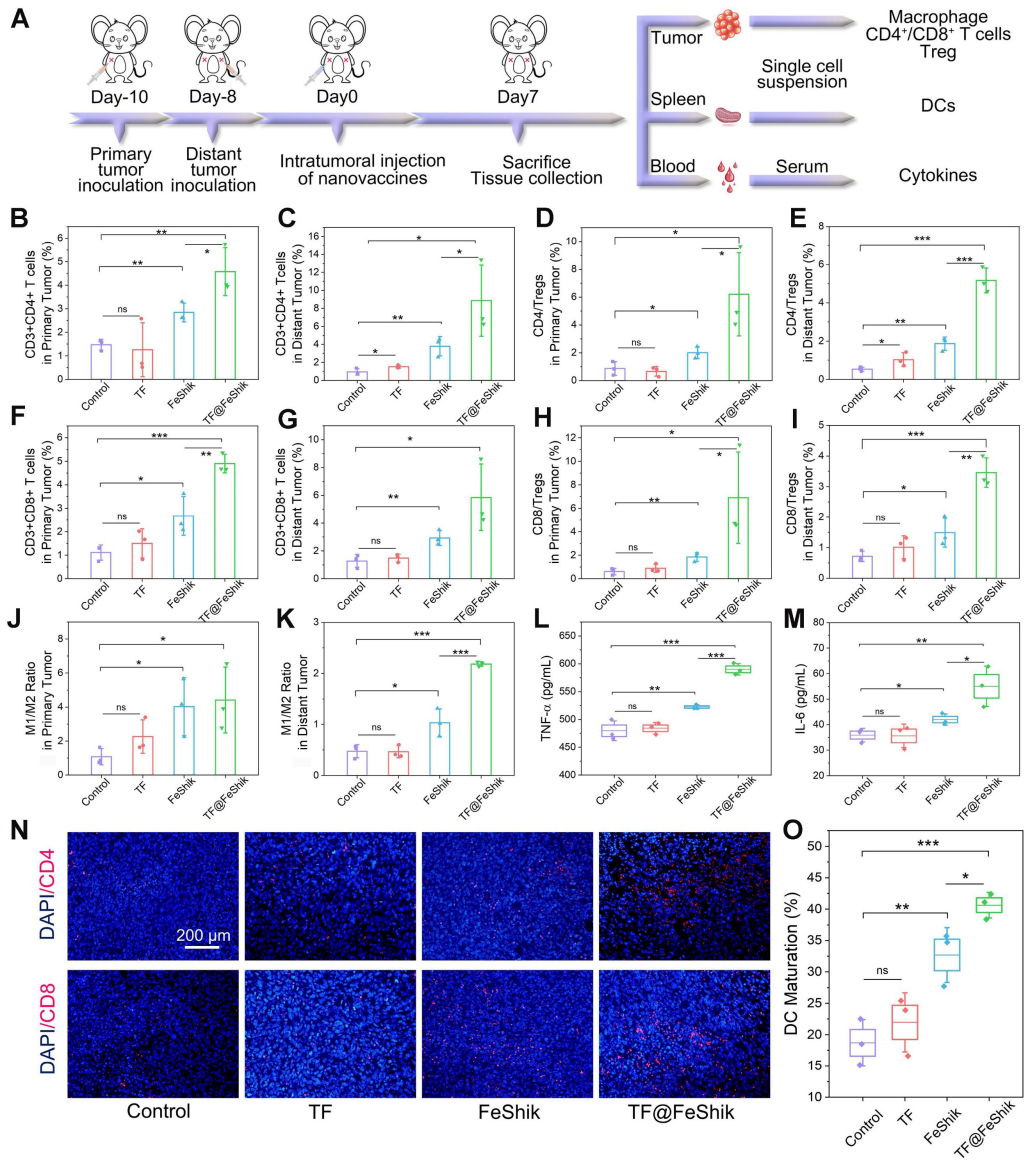
(A) Schematic illustration of the immunity analysis protocol in bilateral 4T1 tumor model. Flow cytometric statistical analysis of the percentages of T helper cells (CD3^+^CD4^+^) in primary tumors (B) and distant tumors (C) after different treatments (n = 3). Flow cytometric statistical analysis of the ratio of T helper cells to Tregs (CD4/Tregs) in primary tumors (D) and distant tumors (E) after different treatments (Treg: CD3^+^CD4^+^Foxp3^+^) (n = 3). Flow cytometric statistical analysis of the percentages of cytotoxic T lymphocytes (CD3^+^CD8^+^) in primary tumors (F) and distant tumors (G) after different treatments (n = 3). Flow cytometric statistical analysis of the ratio of cytotoxic T lymphocytes to Tregs (CD8/Tregs) in primary tumors (H) and distant tumors (I) after different treatments (n = 3). Flow cytometric statistical analysis of the ratio of M1-phenotype macrophages (F4/80^+^CD206^-^CD86^+^) to M2-phenotype macrophages (F4/80^+^CD206^+^CD86^-^) in primary tumors (J) and distant tumors (K) after different treatments (n = 3). Secretion levels of TNF-α (L) and IL-6 (M) in serum after different treatments (n = 3). (N) CD4 and CD8 immunofluorescence staining images of distant tumor tissue slices from mice sacrificed at 14th day after different treatments. (O) Flow cytometric statistical analysis of the DC maturations (CD11c^+^CD80^+^CD86^+^) in spleens after different treatments (n = 3). Data are shown as mean ± SD; n represents the number of biologically independent samples. *p < 0.05, **p < 0.01, and ***p < 0.001.

The immunological analysis is carried out to disclose the underlying mechanism of this abscopal effect (**Figure 6A**). Spleens are collected for the analysis of DC maturation via flow cytometry (**Figure 6O** and** S35**). No obvious difference in DC maturation can be found between TF group and Control. FeShik has a stimulation effect on DC maturation (32.7 ± 3.6%). As expected, the percentage of matured DCs in TF@FeShik group reaches the highest (40.6 ± 1.7%), verifying that the combination of TF antigen and FeShik nanomedicines can effectively amplify the immune responses.

Subsequently, the intratumoral infiltration of CD4^+^ and CD8^+^ T cells are analyzed. For primary tumors, no significant difference can be seen on the frequency of CD4^+^ and CD8^+^ T cells between TF group and Control (**Figure 6B**,**F** and** S36**). The percentages of CD4^+^ and CD8^+^ T cells in FeShik group are obviously increased, reaching 2.84 ± 0.32% and 2.67 ± 0.67%, respectively. TF@FeShik group displays the highest percentages of CD4^+^ (4.57 ± 0.83%) and CD8^+^ (4.90 ± 0.32%) T cells, suggesting that the cytotoxic T lymphocytes have an effective tumor infiltration to enact immune-mediated tumor eradication. Similar results can be seen in distant tumors (**Figure 6C**,**G** and** S37**). FeShik group occupies 3.79 ± 0.87% CD4^+^ T cells and 2.92 ± 0.43% CD8^+^ T cells, which are 4.0-fold and 2.3-fold higher than that in Control. TF@FeShik group occupies 8.85 ± 3.23% CD4^+^ T cells and 5.86 ± 1.96% CD8^+^ T cells, which are 9.4-fold and 4.6-fold higher than that in Control. Remarkably, after treating with TF@FeShik, the CD4^+^/Treg and CD8^+^/Treg ratios are increased by 7.2 times and 11.3 times in primary tumor (**Figure 6D**,**H**), and 9.3 times and 4.8 times in distant tumor (**Figure 6E**,**I**), further indicating that the cold immunosuppressive tumor can be turned into a hot immune activating status by TF@FeShik nanovaccines. Accordingly, the immunofluorescence staining depicts a brighter red fluorescence representing the increased intratumoral infiltration of CD4^+^ or CD8^+^ T cells in FeShik and TF@FeShik groups (**Figure 6N** and** S38**).

Tumor-associated macrophage polarization in bilateral tumors is also studied. Owing to the immunogenicity and ICD effect of TF and FeShik, obvious increases of M1-phenotype macrophages in primary tumors in TF (18.3 ± 2.7%), FeShik (24.9 ± 1.0%), and TF@FeShik (31.1 ± 4.0%) groups can be found compared with Control (14.5 ± 0.3%) (**Figure S39**). Accordingly, the percentages of M2-phenotype macrophages in TF (8.6 ± 1.6%), FeShik (7.0 ± 2.6%), and TF@FeShik (7.6 ± 1.7%) groups are lower than that in Control (15.1 ± 4.6%). As a result, TF@FeShik group exhibits the highest M1/M2 ratio in primary tumors, which is 1.1-fold, 2.0-fold, and 4.1-fold higher than that in FeShik, TF, and Control groups, respectively (**Figure 6J**). TF@FeShik can elevate the M1/M2 ratio in distant tumor as well, which is 4.6-fold and 2.1-fold higher than that in Control and FeShik group (**Figure 6K** and** S40**). However, TF have no effect on tumor-associated macrophage polarization in distant tumors, which is mainly due to the absence of FeShik nanomedicines to stimulate the whole-body immune system. Similar results are also observed on the levels of TNF-α and IL-6 cytokines in serum (**Figure 6L**-**M**), suggesting that FeShik nanomedicines have a critical position in the activation of the antitumor immune responses. All in all, TF@FeShik nanovaccines can stimulate a strong adaptive immunity to inhibit tumor growth in distance.

### Immune memory effect

**Figure 7:**
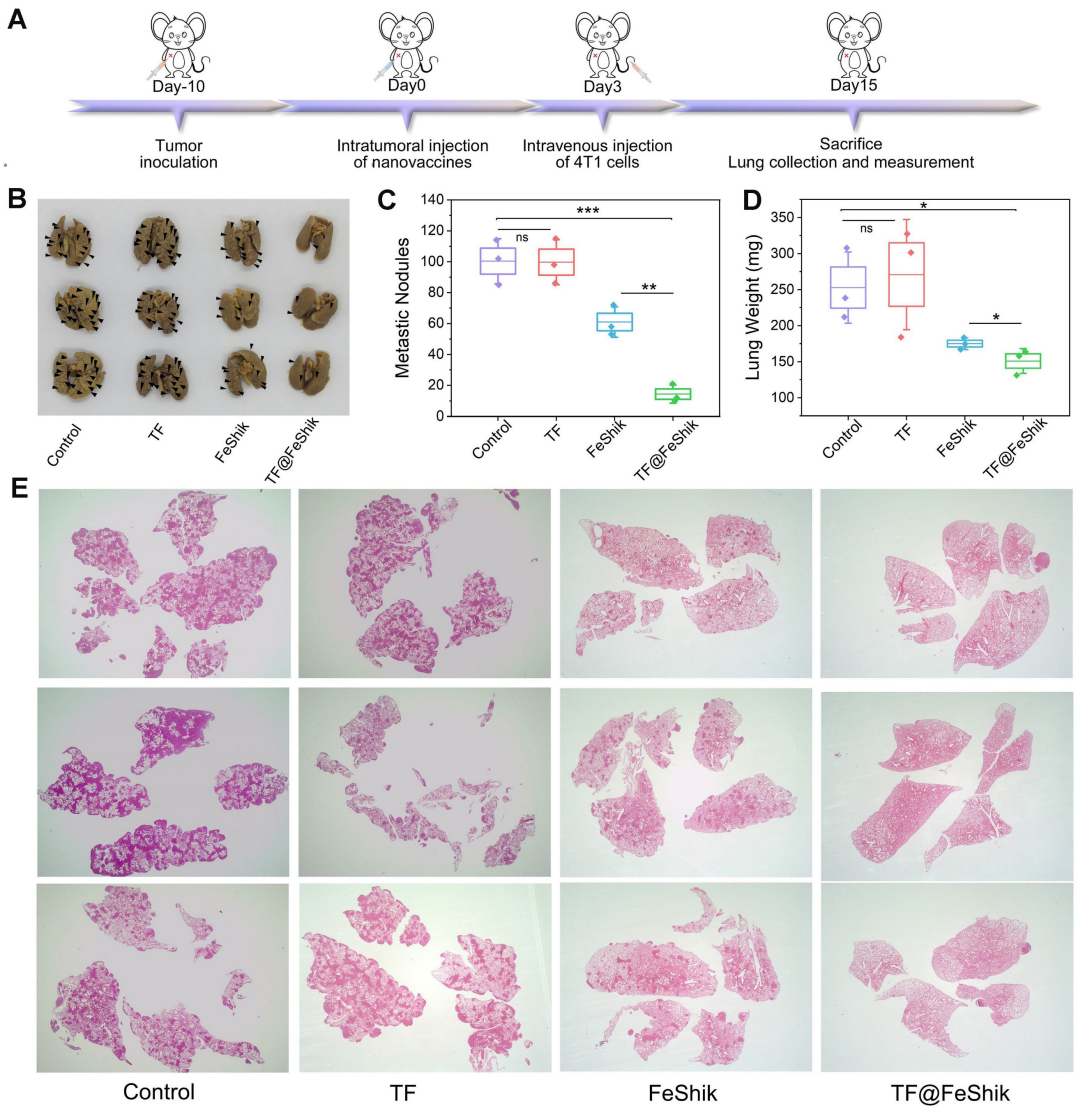
(A) Schematic illustration of the treatment protocol in tumor lung metastasis model. Representative images (B) and summary data (C) of metastasis of lung after different treatments. (D) Average weight of lung after different treatments. (E) H&E images of lungs after different treatments (Magnification × 4). Data are shown as mean ± SD; n represents the number of biologically independent samples. *p < 0.05, **p < 0.01, and ***p < 0.001.

We further evaluate the long-term immune memory effects of TF-FeShik nanovaccines. We first investigate the anti-metastasis effect of TF@FeShik nanovaccines by rechallenging mice bearing 4T1 tumor with 4T1 cells via intravenous injection after various treatments: (i) Control, (ii) TF, (iii) FeShik, (iv) TF@FeShik (**Figure 7A**).** Figure 7B**-**D** shows photographs, metastasis nodules, and weights of lungs in each group. The obvious lung metastasis can be seen in Control and TF group with countless lung metastasis nodules, lung enlargement, and large increase in lung weight. The number of metastasis nodules obviously reduces after FeShik treatment. Surprisingly, the fewest metastasis nodules are found in TF@FeShik group and the corresponding lungs are similar to their normal morphology. H&E staining slices of lung tissues further demonstrate that tumor metastasis is effectually remitted by treating with TF@FeShik nanovaccines (**Figure 7E**).

**Figure 8:**
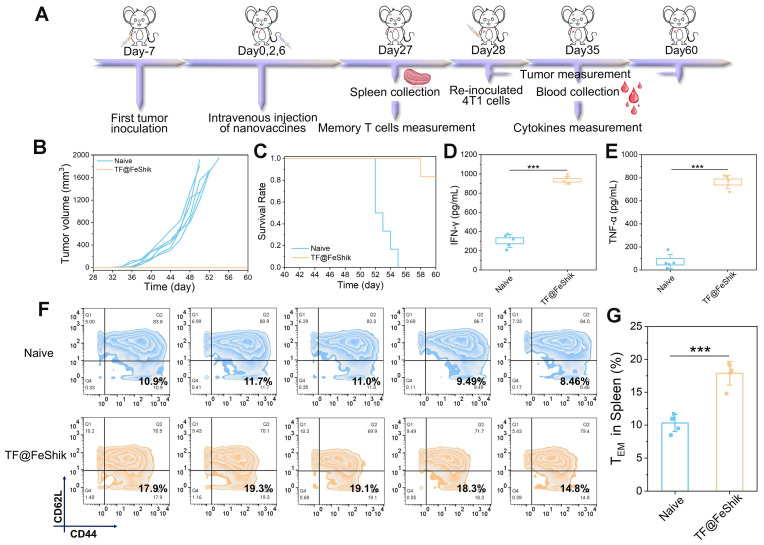
(A) Schematic illustration of the treatment protocol in tumor recurrence model. (B) Individual growth curves of rechallenged tumors in naive mice and TF@FeShik-treated mice (n = 6). (C) Survival curves of naive mice and TF@FeShik-treated mice after 4T1 cell reinoculation (n = 6). IFN-γ (D) and TNF-α (E) levels in serum isolated from naive mice and TF@FeShik-treated mice after 4T1 cell reinoculation (n = 5). Flow cytometric analysis (F) and the corresponding quantification (G) of T_EM_ cells (CD3^+^CD8^+^CD44^+^CD62L^-^) in spleen of splenic lymphocytes in naive mice and TF@FeShik-treated mice before 4T1 cell reinoculation (n = 5). Data are shown as mean ± SD; n represents the number of biologically independent samples. *p < 0.05, **p < 0.01, and ***p < 0.001.

We then investigate the anti-recurrence effect of TF@FeShik nanovaccines. 4T1 tumor-bearing mice are reinoculated with 4T1 cells after TF@FeShik treatment for 28 days (**Figure 8A**). In the meantime, age- and sex-matched naive mice are selected and inoculated with the same number of 4T1 cells as Control. Consequently, mice bearing 4T1 primary tumor with TF@FeShik treatment completely reject tumor recurrence with a promising survival rate of 83.3% at 60th day (**Figure 8B**-**C**). In sharp contrast, tumors in naive mice grow rapidly, and all the naive mice die within 26 days after 4T1 cell inoculation. To better verify the mechanisms underlying the durable immune responses of TF@FeShik nanovaccines, we analyze the frequency of effector memory T cells (CD3^+^CD8^+^CD44^+^CD62L^-^, T_EM_ cells) in spleens before 4T1 cell reinoculation. Intriguingly, the percentage of T_EM_ cells in mice bearing 4T1 tumor with TF@FeShik treatment is 1.7-fold higher than that in naive mice (**Figure 8F**-**G**). TNF-α and interferon-γ (IFN-γ), which are produced by T_EM_ cells upon second encounter with the same antigen 67, increase significantly in mice bearing 4T1 tumor with TF@FeShik treatment after 4T1 cell reinoculation compared to naive mice (**Figure 8D**-**E**). The results above illustrate that TF@FeShik nanovaccines can indeed boost a strong long-term immune memory effect against tumor metastasis and reoccurrence.

### T_1_-weighted MRI imaging

**Figure S41A** shows the T_1_-weighted MRI signals of TF@Feshik in vitro. TF@Feshik possess a concentration-dependent bright enhancement in T_1_-weighted MRI in the absence of GSH. It is noted that the T_1_-weighted MRI signal is significantly enhanced after the addition of GSH, demonstrating the GSH-activated T_1_-weighted MRI capability of TF@Feshik. Accordingly, the T_1_-weighted MRI signal acquired on 4T1 tumor-bearing BALB/c mice exhibit an obvious enhancement after the TF@Feshik treatment (**Figure S41B**). These results suggest that TF@Feshik nanovaccines have great potential to be used as the T_1_-weighted contrast agent for MRI-guided theranostics.

### Biosafety

The biosafety is assessed by injecting various formulations into healthy mice intravenously. There is no visible tissue damage in heart, lungs, liver, spleen, and kidneys under different treatments (**Figure S42**). At the same time, liver and kidney function indexes of treated mice are all located in the normal range (**Figure S43**). Thus, it is confirmed that our TF@FeShik nanovaccines possess great biocompatibility for applications in the future.

## Conclusions

In summary, we have demonstrated on the construction of FeShik supramolecular nanomedicines as ICD stimulants and multifunctional immunoadjuvants for tumor vaccination. The as-prepared FeShik nanomedicines can significantly increase the bioavailability of antigens by prolonging their blood circulation time, facilitating their accumulation in tumor tissues. After phagocytosis by tumor cells, FeShik nanomedicines will disassemble into Fe^2+^ and Shikonin in response to tumor microenvironments, leading to tumor cell ICD via ferroptosis and necroptosis. More importantly, ICD-released autologous tumor cell lysates and pro-inflammatory cytokines not only stimulate DC maturation and antigen cross-presentation, but also promote macrophage repolarization and cytotoxic T lymphocyte infiltration, resulting in the activation of adaptive immune responses toward solid tumors. Thanks to the multifunctionality of FeShik nanomedicines, nanovaccines based on FeShik nanomedicines exhibit an enhanced eradication effect on primary tumors, an amplified abscopal effect to inhibit distant tumor growth, and a strong long-term immune memory effect against tumor metastasis and reoccurrence. Encouraged by the diversity of polyphenols and metal ions, our research may provide a valuable paradigm to establish a large library for tumor vaccination.

## Methods

### Chemicals and Reagents

Shikonin was purchased from Chengdu Gelipu Biotechnology Co., Ltd. Ovalbumin (OVA) and IR780 were obtained from Sigma-Aldrich. Iron chloride hexahydrate (FeCl_3_·6H_2_O), Glutathione (GSH), 1,10-phenanthroline, and 5,5-dimethyl-1-pyrroline N-oxide (DMPO) were purchased from Aladdin. FITC was obtained from Solarbio. Hydrogen peroxide (H_2_O_2_) was obtained from Beijing Chemical Works. Disodium terephthalate (TPA) was bought from Alfa Aesar. Every Green Fetal bovine serum (FBS) was purchased from Zhejiang Tianhang Biotechnology Co., Ltd. PBS, RMPI-1640 basic, and DMEM were purchased from Gibco. Cell counting kit-8 (CCK-8) was obtained from Bimake. Hoechst 33342, Lyso-Tracker Red, Hanks' Balanced Salt Solution, Reactive oxygen species assay kit, ATP assay kit, Coomassie Blue Staining Kit, and BCA protein assay kit were obtained from Beyotime. Ferrostatin-1 (Fer-1), necrostatin-1 (Nec-1), AC-DEVD-CHO (APO), and deferoxamine (DFO) were purchased from Selleck Chemicals. FerroOrange was obtained from Dojindo. GSH assay kit was bought from Nanjing Jiancheng. Anti-Glutathione Peroxidase 4 (GPX4) antibody, anti-receptor-interacting serine/threonine-protein kinase 1 (RIP1) antibody, anti-Calreticulin (CRT) antibody, and anti-HMGB1 antibody were purchased from Abcam. Anti-RIP3 antibody was purchased from Proteintech. GPX4, RIP1, RIP3, tumor necrosis factor α (TNF-α), interferon-γ (IFN-γ) ELISA assay kit were bought from Spbio. BODIPY^581/591^-C11 was bought from Thermo Fisher. Goat Anti-rabbit IgG H&L/FITC antibody was bought from Bioss. Annexin-V-FITC apoptosis analysis kit was purchased from Sungene Biotech. Anti-CD11c-FITC, Anti-CD80-PE, CD86-PE-Cyanine5, OVA (SIINFEKL) peptide bound to H-2Kb antibody-PE, MHC-I-PE, CD3-FITC, CD4-PE, CD8a-PE-Cyanine5, Foxp3-PE-Cyanine5, F4/80-FITC, CD206-PE, CD62L-APC, and CD44-PE were purchased from eBioscience. Interleukin-6 (IL-6) ELISA assay kit was purchased from Shanghai Tongwei Biotechnology Co., Ltd. Bouin Fixative Solution was purchased from XinFan Bio-technology Co., Ltd. 4% Paraformaldehyde (PFA) solution was purchased from Biosharp.

### Animals

Female BALB/c mice (8 weeks, 18~20 g) were purchased from Beijing Vital River Laboratory Animal Technology. All animal experiments were performed following the guidelines of Laboratory Animals of the First Hospital of Jilin University and approved by the Animal Laboratory Ethics Committee (No: 0693).

### Characterization

Transmission electron microscopy (TEM) images were acquired on a JEOL JEM-2100F field emission electron microscope. Dynamic diameters and zeta potentials were assessed by a Malvern Zetasizer Nano ZS. UV-vis absorption spectra were detected by a Shimadzu 2600 UV-vis-NIR spectrophotometer. Fourier transform infrared (FTIR) spectra were determined by Bruker VERTEX 80V. Photoluminescence (PL) spectroscopy was obtained on a Shimadzu RF-6000 fluorescence spectrometer. Electron spin resonance (ESR) spectra were recorded on Brucker ELEXSYS spectrometer. OLYMPUS FV1000 was used to detect the confocal laser scanning microscopy (CLSM) images. Flow cytometry was performed by BD FACSCalibur Flow Cytometer. The fluorescence images of the mice were obtained on a two-dimensional InGaAs array (Princeton Instruments, NIRvana-640). UHPLC-MS/MS analyses were performed using a nanoElute UHPLC system (Bruker, Germany) coupled with a tims TOF pro2 mass spectrometer (Bruker, Germany) in Novogene Co., Ltd.

### Statistical methods

All experimental statistical values were presented as means ± SD. Student's t-test was used to analyze the differences between two groups, ***p < 0.001, **p < 0.01 and *p < 0.05.

### Preparation of OVA@FeShik

30 mg OVA was dissolved in 30 mL ultrapure water. FeCl_3_ solution (10 mg/mL in ultrapure water) and Shikonin (5 mg/ mL in ethanol) were successively added into OVA solution under vortex for 30 s. After 2.5 h, the mixing solution was concentrated and washed with ultrapure water by ultrafiltration Millipore tube (100 kDa) for more than 3 times. The acquired solution was diluted to an appropriate concentration for further use.

### GSH responsive

OVA@FeShik was treated with or without 10 mM GSH in dialysis bags under shaking. At different timepoints, 3 mL supernatant was removed and replaced with new medium. As for Shikonin release, the absorbance value of supernatant at 517 nm was detected by UV-vis absorption spectrometer, and the release amount of Shikonin was calculated by drawing a standard curve. For the detection of ferrous ions, 1,10-phenanthroline was used as an indicator added into the supernatant, and the supernatant before the addition of 1,10-phenanthroline was used as the background solution. The amount of ferrous iron released was calculated by measuring their absorbance values at 510 nm.

### •OH Generation

Firstly, 20 μM TPA was used as a probe to detect the generation of •OH by fluorescence spectrometer. Briefly, GSH pre-treated OVA@FeShik was incubated with TPA and H_2_O_2_. The Fluorescence spectrum (excitation 310 nm) of mixing solution was studied. In addition, 100 μM DMPO was used as a trapping agent to determine •OH by ESR spectrometer.

### Cytotoxicity assay

Cytotoxicity assay of OVA@FeShik or OVA toward 4T1 cells and L929 cells were studied through CCK-8 assay. Cells were seeded in 96-well culture plates and incubated with 10% FBS culture medium for 24 h. OVA@FeShik or OVA in different concentrations were added and incubated for 24 h. Finally, 10 μL CCK-8 was added into each well. After a period of time, the OD value at 450 nm of each well was measured by a microplate reader.

As for the cytotoxicity assay of OVA@FeShik toward 4T1 cells with different inhibitors, Fer-1, APO, or Nec-1 was added into 60 μg/mL OVA@FeShik treated cells and the cytotoxicity assay was studied.

### Cellular uptake and localization assay

First, ^FITC^OVA was prepared. 30 mg OVA and 3 mg FITC were added into 15 mL ultrapure water under stirring for 10 h in dark. Then, the mixing solution was isolated and purified by dialysis (8000 Da) for more than 24 h. The preparation of ^FITC^OVA@FeShik is similar to the OVA@FeShik, in which OVA was replaced with ^FITC^OVA. 4T1 cells were seeded in 2 cm confocal dishes and incubated with 10% FBS RMPI-1640 for 24 h. Then, ^FITC^OVA or ^FITC^OVA@FeShik (at an equivalent dosage of 60 μg/mL OVA@FeShik) was added and incubated with 4T1 cells. At different timepoints, the dishes were washed with HBSS for 3 times, and incubated with Lyso-Tracker Red medium (75 nM in HBSS) at 37 °C for 15 min. Subsequently, they were washed twice and assessed on CLSM.

### Intracellular ROS level assay

4T1 cells were treated with OVA, FeShik, OVA@FeShik (at an equivalent dosage of 60 μg/mL OVA@FeShik) or without any treatment as the Control for 24 h in confocal dishes. After being washed with PBS, the cells were incubated with DCFH-DA (10 μM in RMPI-1640 basic) for 20 min. Next, the cells were washed and the fluorescence images of cells in different groups were acquired by CLSM.

4T1 cells were treated with FeShik, OVA@FeShik (at an equivalent dosage of 60 μg/mL OVA@FeShik) or without any treatment as the Control for 24 h in 24-well plates. After that, the cells were collected and washed with PBS. Next, the cells were stained with DCFH-DA (10 μM in RMPI-1640 basic) for 20 min. And then, the cells were washed and the fluorescent signals representing the ROS level were acquired by flow cytometry.

### Intracellular ferrous ion level assay

4T1 cells were treated with OVA, FeShik, OVA@FeShik (at an equivalent dosage of 60 μg/mL OVA@FeShik), or without any treatment as the Control for 24 h in confocal dishes. After being washed with HBSS, the cells were incubated with FerroOrange (1 μM in HBSS) for 30 min. Next, the fluorescence images of cells in different groups were acquired by CLSM.

### Intracellular GSH content assay

4T1 cells were treated with OVA, FeShik, OVA@FeShik (at an equivalent dosage of 60 μg/mL OVA@FeShik), or without any treatment as the Control for 24 h in 24-well plates. After that, the cells were collected and lysed. Next, the supernatant was collected by centrifugation and the GSH content in different groups were assessed according to the reduced GSH assay kit.

### Intracellular LPO assay

4T1 cells were treated with OVA, FeShik, OVA@FeShik (at an equivalent dosage of 60 μg/mL OVA@FeShik), or without any treatment as the Control for 24 h in confocal dishes. After being washed with HBSS, the cells were incubated with BODIPY^581/591^-C11 (10 μM in HBSS) for 30 min. Next, the cells were washed and the fluorescence images of cells in different groups were acquired by CLSM.

### Intracellular expression of GPX4, RIP1, and RIP3 assay

4T1 cells were treated with OVA, FeShik, OVA@FeShik (at an equivalent dosage of 60 μg/mL OVA@FeShik), or without any treatment as the Control for 24 h in confocal dishes. After being washed with PBS and fixed with 4% PFA, the cells were treated with immunostaining permeabilization buffer for 10 min. Next, the cells were washed and blocked with blocking buffer. Subsequently, the cells were incubated with anti-GPX4, RIP1, or RIP3 primary antibody overnight at 4 °C. And then, the cells were washed and incubated with IgG H&L/FITC secondary antibody for 1 h. The cells were washed and treated with Hoechst 33342 to stain the cell nucleus. Finally, the fluorescence images of cells in different groups were acquired by CLSM.

4T1 cells were treated with OVA, FeShik, OVA@FeShik (at an equivalent dosage of 60 μg/mL OVA@FeShik), or without any treatment as the Control for 24 h in 6-well plates. After that, the cells were collected and lysed. Next, the supernatant was collected by centrifugation and the expression of GPX4, RIP1, or RIP3 in different groups were assessed according to the ELISA assay kit.

4T1 cells were treated with OVA, FeShik, OVA@FeShik (at an equivalent dosage of 60 μg/mL OVA@FeShik), or without any treatment as the control for 24 h in 6-well plates. After that, the cells were collected and lysed. Next, the protein supernatant was collected by centrifugation and the expression of GPX4, RIP1, or RIP3 in different groups were assessed by western blot. Proteins were separated via a 12% SDS-polyacrylamide gel electrophoresis and transferred to PVDF membranes. The PVDF membranes were cropped at different molecular weights according to the color protein marker. After that, they were blocked with blocking buffer. And then, they were incubated with anti-GAPDH antibody, anti-GPX4 antibody, anti-RIP1 antibody, or anti-RIP3 antibody at 4 °C overnight. Finally, they were probed with secondary antibodies and tested according to the ECL kit.

### Cell necroptosis assessment

4T1 cells were treated with OVA@FeShik at different concentrations for 24 h in 24-well plates. After that, the cells were collected and washed with PBS. Next, the cells were stained and the necroptosis assessment was acquired by flow cytometry.

### Expression of CRT assay by immunofluorescence analysis and ELISA test

4T1 cells were treated with OVA, FeShik, OVA@FeShik (at an equivalent dosage of 60 μg/mL OVA@FeShik), or without any treatment as the Control for 24 h in confocal dishes. After being washed with PBS and fixed with 4% PFA, the cells were treated with immunostaining permeabilization buffer for 10 min. Next, the cells were washed and blocked with blocking buffer. Subsequently, the cells were incubated with anti-CRT primary antibody overnight at 4 °C. And then, the cells were washed and incubated with IgG H&L/FITC secondary antibody for 1 h. The cells were washed and treated with Hoechst 33342 to stain the cell nucleus. Finally, the fluorescence images of cells in different groups were acquired by CLSM.

4T1 cells were treated with OVA@FeShik at different concentrations for 24 h in 24-well plates. After that, the cells were collected and washed with PBS. Next, the cells were incubated with anti-CRT primary antibody for 30 min. And then, the cells were washed with PBS and treated with IgG H&L/FITC secondary antibody for 30 min. After being washed with PBS, the fluorescent signals representing the expression of CRT level were acquired by flow cytometry.

### Expression of HMGB1 assay by immunofluorescence analysis and ELISA test

4T1 cells were treated with OVA, FeShik, OVA@FeShik (at an equivalent dosage of 60 μg/mL OVA@FeShik), or without any treatment as the Control for 24 h in confocal dishes. After being washed with PBS and fixed with 4% PFA, the cells were treated with immunostaining permeabilization buffer for 10 min. Next, the cells were washed and blocked with blocking buffer. Subsequently, the cells were incubated with anti-HMGB1 primary antibody overnight at 4 °C. And then, the cells were washed and incubated with IgG H&L/FITC secondary antibody for 1 h. The cells were washed and treated with Hoechst 33342 to stain the cell nucleus. Finally, the fluorescence images of cells in different groups were acquired by CLSM.

### Expression of CRT and HMGB1 assay by western blot measurement

4T1 cells were treated with OVA, FeShik, OVA@FeShik (at an equivalent dosage of 60 μg/mL OVA@FeShik), or without any treatment as the control for 24 h in 6-well plates. After that, the cells were collected and lysed. Next, the protein supernatant was collected by centrifugation and the expression of CRT or HMGB1 in different groups were assessed by western blot. Proteins were separated via a 12% SDS-polyacrylamide gel electrophoresis and transferred to PVDF membranes. The PVDF membranes were cropped at different molecular weights according to the color protein marker. After that, they were blocked with blocking buffer. And then, they were incubated with anti-GAPDH antibody, anti-CRT antibody, or anti-HMGB1 antibody at 4 °C overnight. Finally, they were probed with secondary antibodies and tested according to the ECL kit.

### ATP secretion levels assay

4T1 cells were treated with OVA@FeShik at different concentrations for 24 h in 6-well plates. After that, the medium in different groups was collected and analyzed by using ATP assay kit.

### In vitro DC maturation and antigen cross-presentation

BMDCs were collected from the mice bone marrow of healthy BALB/c mice. The hind legs of BALB/c mice were cut away and the muscle on the hind legs was moved away. After being steeped in 70% ethanol, the end of the bone was cut away. Next, 1 mL syringe filled with HBSS was inserted into the bone and red marrow in the bone was flushed out and collected until the bone became white. After that, the cells were collected by centrifugation and the red blood cell was lysed by lysis buffer. Subsequently, the cells were cultured with RMPI-1640 basic containing 20 ng/mL GM/CSF, 10% FBS, 20 mM HEPES, 1X non-essential amino acids, 55 μM 2-Hydroxy-1-ethanethiol, 50 U/mL penicillin-streptomycin, 1 mM sodium pyruvate, and 2 mM glutamine for 7 days. The suspension cells were collected as BMDCs, and the adherent cells were BMDMs. 4T1 cells was seeded in the 24-well plates and treated with 60 μg/mL ^FITC^OVA@FeShik for 24 h. After that, 4T1 cells were transferred into the upper chamber in a Transwell system, and the BMDCs or BMDMs were seeded in the lower chamber and co-incubated with 4T1 tumor cells. Next, the lower chamber cells were harvested and washed. After being stained with Hoechst 33342, the fluorescence images of cells in different groups were acquired by CLSM. As for the study of DC maturation, the 4T1 cells were treated with OVA, FeShik, OVA@FeShik (at an equivalent dosage of 60 μg/mL OVA@FeShik), or without any treatment as the Control for 24 h. After that, the BMDCs were co-incubated with 4T1 tumor cells. And then, the cells were harvested and stained with anti-CD11c-FITC, anti-CD80-PE, and anti-CD86-PE-cyanine5. After being washed with PBS, the fluorescence signals of cells were acquired by flow cytometry. As for the study of antigen cross-presentation, the cells were harvested and stained with anti-CD11c-FITC, anti-H2Kb-SIINFEKL-PE, and anti-CD86-PE-cyanine5 or stained with anti-CD11c-FITC, anti-MHC-I-PE, and anti-CD86-PE-cyanine5. After being washed with PBS, the fluorescence signals of cells were acquired by flow cytometry.

### Pharmacokinetics assay in vivo

Firstly, ^IR780^OVA was prepared. 30 mg OVA and 45 μL of 10 mg/mL IR780 DMSO solution were added into 15 mL ultrapure water under stirring at 70 °C for 2 h in dark. After that, the mixing solution was concentrated and washed with ultrapure water by ultrafiltration Millipore tube (10 kDa) for more than 3 times and the ^IR780^OVA was successfully prepared. The preparation of ^IR780^OVA@FeShik is similar with the OVA@FeShik, in which OVA was replaced with ^IR780^OVA. As for the pharmacokinetics assay, nine female BALB/c mice were injected intravenously with ^IR780^OVA, ^IR780^FeShik, and ^IR780^OVA@FeShik, respectively. Blood was collected into the anticoagulant tube from the orbital venous plexus of mice by using a capillary pipette at different timepoints. And then, the fluorescence intensity corresponding to IR780 was measured on a two-dimensional InGaAs array (Princeton Instruments, NIRvana-640) with a laser wavelength of 808 nm.

### Biodistribution assay

Firstly, orthotopic 4T1 tumor model was constructed on female BALB/c mice. And then, they were divided into 3 groups and injected intravenously with ^IR780^OVA,^ IR780^FeShik, and ^IR780^OVA@FeShik, respectively. The fluorescence images were measured by scanning the whole body of mice on a two-dimensional InGaAs array (Princeton Instruments, NIRvana-640) with a laser wavelength of 808 nm.

### In vivo antitumor effect and antitumor immune mechanism study

Firstly, orthotopic 4T1 tumor model was constructed on female BALB/c mice. When tumors reached ~50 mm^3^, mice were randomly divided into 4 groups (n = 9) and injected intravenously with (ii) OVA, (iii) FeShik, (iv) OVA@FeShik, and (i) without any treatment as the Control. Various agents with the equivalent dosage related to 5 mg/kg FeShik. The length and width of each tumor and the body weight were recorded every 2 days. After 14 days, the mice in each group were sacrificed to collect blood, tumors, and spleens. 3 tumors in each group were photographed to compare the antitumor effect of different groups.

As for the histopathology and immunohistochemistry analyses, tumor tissues were stained and examined on fluorescence microscope. In brief, the histopathology analysis of tumor section was performed by hematoxylin-eosin (H&E) staining. For the expression levels of GPX4, RIP1, and RIP3, anti-GPX4, anti-RIP1, and anti-RIP3 antibodies were used as the primary antibody and IgG H&L/FITC antibody was used as the secondary antibody. Anti-CD4-PE antibody and anti-CD8a-PE-Cyanine5 were used to analyze the expression of CD4^+^ and CD8^+^ T cells in tumor section.

As for the DCs, macrophage, and T cells measurements, flow cytometry was used. Firstly, the single-cell suspension of spleens was prepared to test the DC maturation after being stained with anti-CD11c-FITC, anti-CD80-PE, and anti-CD86-PE-cyanine5. Secondly, the phenotype of macrophages was studied by preparing the single-cell suspension of tumors, and they were stained with F4/80-FITC, CD206-PE, and CD86-PE-Cyanine5. Thirdly, the T helper cells and cytotoxic T lymphocytes in single-cell suspension of tumors were tested by using anti-CD3-FITC, CD4-PE, and CD8a-PE-Cyanine5.

As for the cytokine detection, the blood obtained from each group was centrifuged to collect serum. The secretion levels of IL-6 and TNF-α in the serum were tested according to the ELISA assay kits.

### Preparation of TF@FeShik

Firstly, the TF was prepared and used as the homologous antigen. 4T1 cells were collected and dispersed in water, and they were immediately frozen in liquid nitrogen, followed by four freeze-thaw cycles. After centrifugation, the supernatant was collected and 4T1 TF was obtained. The protein composition of extracted TF is characterized by UHPLC-MS/MS analyses. The resulting spectra were searched against Mus_musculus_uniprot_2023_3_13.fasta (88040 sequences) database by the search engines: MaxQuant (Bruker, Tims). The TF content was tested by BCA protein assay kit. The preparation of TF@FeShik is similar with the OVA@FeShik, in which OVA was replaced by TF.

### Protein characterization by Coomassie Brilliant Blue staining

The protein profiles of TF, FeShik, and TF@FeShik were studied by Coomassie Brilliant Blue staining. Firstly, the protein amounts in all samples were examined according to BCA protein assay kit. And then, all samples were dispersed in loading buffer and heated to 100 °C for 20 min. Subsequently, they were fractionated by SDS-PAGE. After that, protein was stained by Coomassie Blue for 4 h. Finally, they were destained and imaged to observe the protein profiles.

### In vivo antitumor effect in a bilateral 4T1 tumor model

4T1 cells were kindly provided from Changchun Institute of Applied Chemistry Chinese Academy of Sciences. Bilateral orthotopic 4T1 tumor model was constructed on female BALB/c mice. 4T1 cells were orthotopic injected into BALB/c mice at the right mammary fat pads (1.0×10^6^ cells per mouse) as primary tumors. After 2 days, 4T1 cells were orthotopic injected into BALB/c mice at the left mammary fat pads (5.0×10^5^ cells per mouse) as distant tumors. After 8 days, mice were randomly divided into 4 groups (n = 15) and injected with (ii) TF, (iii) FeShik, (iv) TF@FeShik, and (i) without any treatment as the Control into primary tumors. Various agents with the equivalent dosage related to 5 mg/kg FeShik. The length and width of each tumor and the body weight of each mouse were recorded every 2 days. After 14 days, five mice in each group were sacrificed to collect tumors. The tumors in each group were photographed and weighed to compare the antitumor effect of different groups. In addition, the histopathology and immunohistochemistry analyses including the H&E staining and the expression levels of GPX4, RIP1, RIP3, CRT, and HMGB1 were performed to investigate the underlying mechanism. In addition, the survival rate of mice is monitored by recording the survival of mice (n = 10) in each group every day.

### In vivo immune responses in a bilateral 4T1 tumor model

Bilateral orthotopic 4T1 tumor model was constructed on female BALB/c mice. 4T1 cells were inoculated into BALB/c mice at the right mammary fat pads (1.0×10^6^ cells per mouse) as primary tumors. After 2 days, 4T1 cells were inoculated into BALB/c mice at the left mammary fat pads (5.0×10^5^ cells per mouse) as distant tumors. After 8 days, mice were randomly divided into 4 groups (n = 3) and injected with (ii) TF, (iii) FeShik, (iv) TF@FeShik, and (i) without any treatment as the Control into primary tumors. Various agents with the equivalent dosage related to 5 mg/kg FeShik. After 7 days, the mice in each group were sacrificed to collect blood, spleens, and tumors. Blood was used to collect serum by centrifugation and cytokines (TNF-α and IL-6) in serum were tested according to the ELISA assay kits. Spleens and tumors were used to obtain a single-cell suspension. DC maturation, the phenotype of macrophages, the T helper cells, cytotoxic T lymphocytes, and Tregs were determined by flow cytometry. For immunohistochemistry analyses, primary and distant tumors in each group were fixed and stained to analyze the expression of CD4 and CD8.

### Anti-metastasis studies

Orthotopic 4T1 tumor model was constructed on female BALB/c mice. 4T1 cells were inoculated into BALB/c mice at the right mammary fat pads (1.0×10^6^ cells per mouse). After 10 days, mice were randomly divided into 4 groups (n = 5) and injected with (ii) TF, (iii) FeShik, (iv) TF@FeShik, and (i) without any treatment as the Control. Various agents with the equivalent dosage related to 5 mg/kg FeShik. After 3 days, 1.0×10^6^ 4T1 cells were intravenously injected into mice. After 12 days, the mice in each group were sacrificed to collect lungs. And then, the lungs were fixed and stained with bouin fixative solution and photographed. The lung morphology was observed and lung nodules were counted. The lungs in each group were weighed. In addition, the histopathology analysis of lung section was performed by H&E staining.

### Anti-recurrence studies

Orthotopic 4T1 tumor model was constructed on female BALB/c mice. 4T1 cells were inoculated into BALB/c mice at the right mammary fat pads (5.0×10^5^ cells per mouse on day -7). Mice were injected with 10 mg/kg TF@FeShik on day 0, day 2, and day 6. On day 27, spleens of 5 mice after treating with TF@FeShik and 5 naive mice were used to obtain a single-cell suspension. T_EM_ cells were determined by flow cytometry. On day 28, mice after treating with TF@FeShik and naive mice were inoculated with 1.0×10^6^ 4T1 cells. The length and width of each tumor were recorded every 2 days. On day 35, blood was used to collect serum by centrifugation and cytokines (TNF-α and IFN-γ) in serum were tested according to the ELISA assay kits. As for counting survival rate in different group, mice fed for 60 days. Considering ethical issues, mice were euthanized when the size of tumor exceeded 2000 mm^3^.

### T_1_-weighted MRI signals studies

For T_1_-weighted MRI signals studies in vitro, TF@FeShik solution at different concentrations was treated with or without GSH to test the T_1_-weighted signals. As for T_1_-weighted MRI signals studies in vivo, 4T1 tumor-bearing BALB/c mice were intravenously injected with TF@FeShik. The T_1_-weighted MRI signals were acquired after 24 h.

### Biosafety studies

Healthy female BALB/c mice were randomly divided into 6 groups (n = 3) and injected with (ii) OVA, (iii) TF, (iv) FeShik, (v) OVA@FeShik, (vi) TF@FeShik, and (i) without any treatment as the Control. Various agents with the equivalent dosage related to 5 mg/kg FeShik. After 7 days, the mice in each group were sacrificed to collect blood and major organs. Blood was used to collect serum by centrifugation and liver/kidney function indexes in serum were tested. Major organs in each group are fixed in 4% formaldehyde at room temperature for H&E staining.

## Supplementary Material

Supplementary figures.

## Abbreviations

FeShik: Fe^3+^-Shikonin metal-phenolic networks
ICD: immunogenic cell death
T helper type-2: Th2
TLR: toll-like receptor
ROS: reactive oxygen species
RIP1: receptor-interacting protein kinase-1
RIP3: receptor-interacting protein kinase-3
MLKL: mixed lineage kinase domain-like
OVA: Ovalbumin
TF: tumor cell fragment
GSH: Glutathione
H_2_O_2_: hydrogen peroxide
GPX4: glutathione peroxidase 4
TEM: transmission electron microscopy
FTIR: fourier transform infrared
PL: photoluminescence
ESR: electron spin resonance
CLSM: confocal laser scanning microscopy
DMPO: 5,5-dimethyl-1-pyrroline N-oxide
CCK-8: cell counting kit-8
FITC: fluorescein isothiocyanate
DCFH-DA: 2',7'-dichlorofluorescin diacetate
CRT: calreticulin
HMGB1: high-mobility group protein B1
ATP: adenosine triphosphate
ELISA: enzyme-linked immunosorbent assay
BMDCs: bone-marrow-derived dendritic cells
NIR: near-infrared
H&E: hematoxylin-eosin
TNF-α: tumor necrosis factor α
IFN-γ: interferon-γ
IL-6: interleukin-6
T_EM_ cells: effector memory T cells
