# Supplementary material for: Fe(III)-Shikonin supramolecular nanomedicines as immunogenic cell death stimulants and multifunctional immunoadjuvants for tumor vaccination
Source: Theranostics. 2023 Sep 25;13(15):5266–89. doi: 10.7150/thno.81650 (PMC10614674; doi:10.7150/thno.81650)

Supporting Information

Fe(III)-Shikonin supramolecular nanomedicines as immunogenic cell death stimulants and multifunctional immunoadjuvants for tumor vaccination

Wenjie Feng,[1] Wanrui Shi,[2] Yanqi Cui,[2] Jiajun Xu,[2] Shuwei Liu,[1] Hang Gao,[2] Shoujun Zhu,[1,2] Yi Liu,[2,] and Hao Zhang[1,2,3,]**

[1] Joint Laboratory of Opto-Functional Theranostics in Medicine and Chemistry, The First Hospital of Jilin University, Changchun 130021, P. R. China

[2] State Key Laboratory of Supramolecular Structure and Materials, College of Chemistry, Jilin University, Changchun 130012, P. R. China

[3] Green Catalysis Center, College of Chemistry, Zhengzhou University, Zhengzhou 450001, P. R. China

*E-mail addresses: yiliuchem@jlu.edu.cn; hao_zhang@jlu.edu.cn

Figure S1. Hydrated diameter of FeShik.

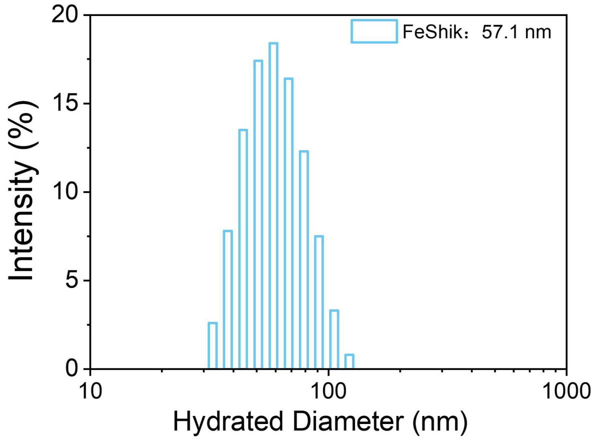

Figure S2. Standard absorption curve of OVA according to the concentration and UV-vis absorbance at 269 nm.

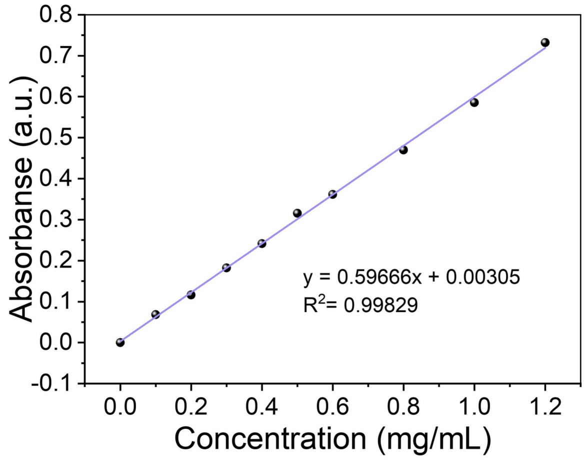

Figure S3. Quantification of Fe^{2+} by using 1,10-phenanthroline as the indicator. UV-vis absorption spectra of 1,10-phenanthroline after treating with Fe^{2+} at different concentrations (A) and the standard absorption curve of Fe^{2+} according to the concentration and UV-vis absorbance at 510 nm (B).

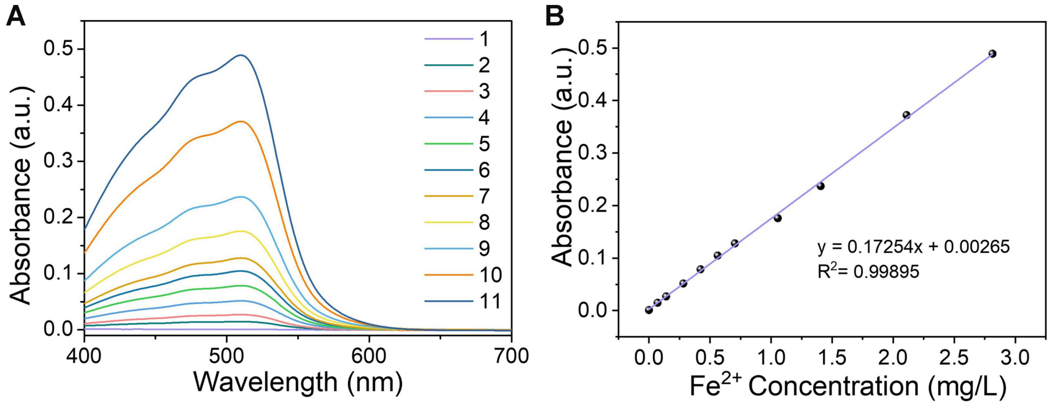

Figure S4. UV-vis absorption spectra of Shikonin at different concentrations (A) and the standard absorption curve of Shikonin according to the concentration and UV-vis absorbance at 517 nm (B).

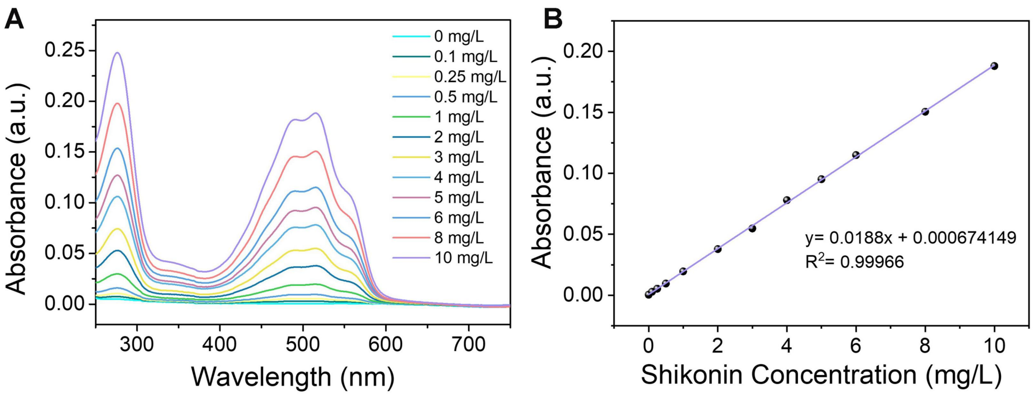

Figure S5. Physiological stability of OVA@FeShik in H_2O, PBS, and 1640 basic with or without 10% FBS before (A) and after (B) 7 days. (C) Time-dependent hydrated diameter of OVA@FeShik in H_2O, PBS, and 1640 basic with 10% FBS (n = 3). Data are shown as mean ± SD; n represents the number of independent samples.

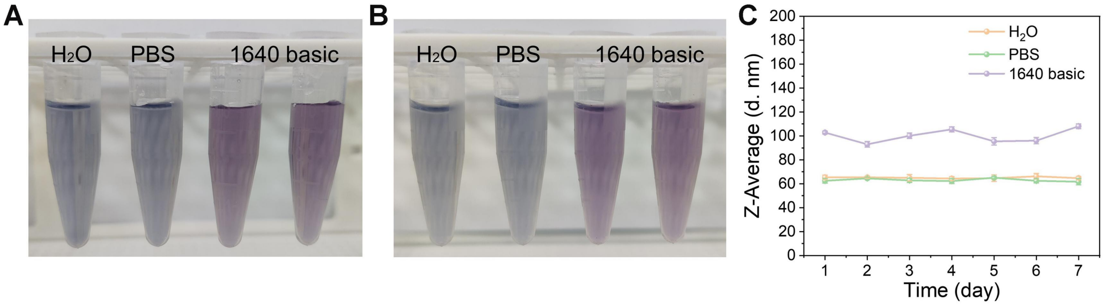

Figure S6. Cell viability of 4T1 cells (A) and L929 cells (B) after treating with different concentrations of OVA@FeShik (n = 5). GSH level of 4T1 cells (C) and L929 cells (D) after treating with different concentrations of OVA@FeShik (n = 3). Cell viability of 4T1 cells (E) and L929 cells (F) after treating with different concentrations of OVA (n = 5). Data are shown as mean ± SD; n represents the number of biologically independent samples. *p < 0.05, **p < 0.01, and ***p < 0.001.

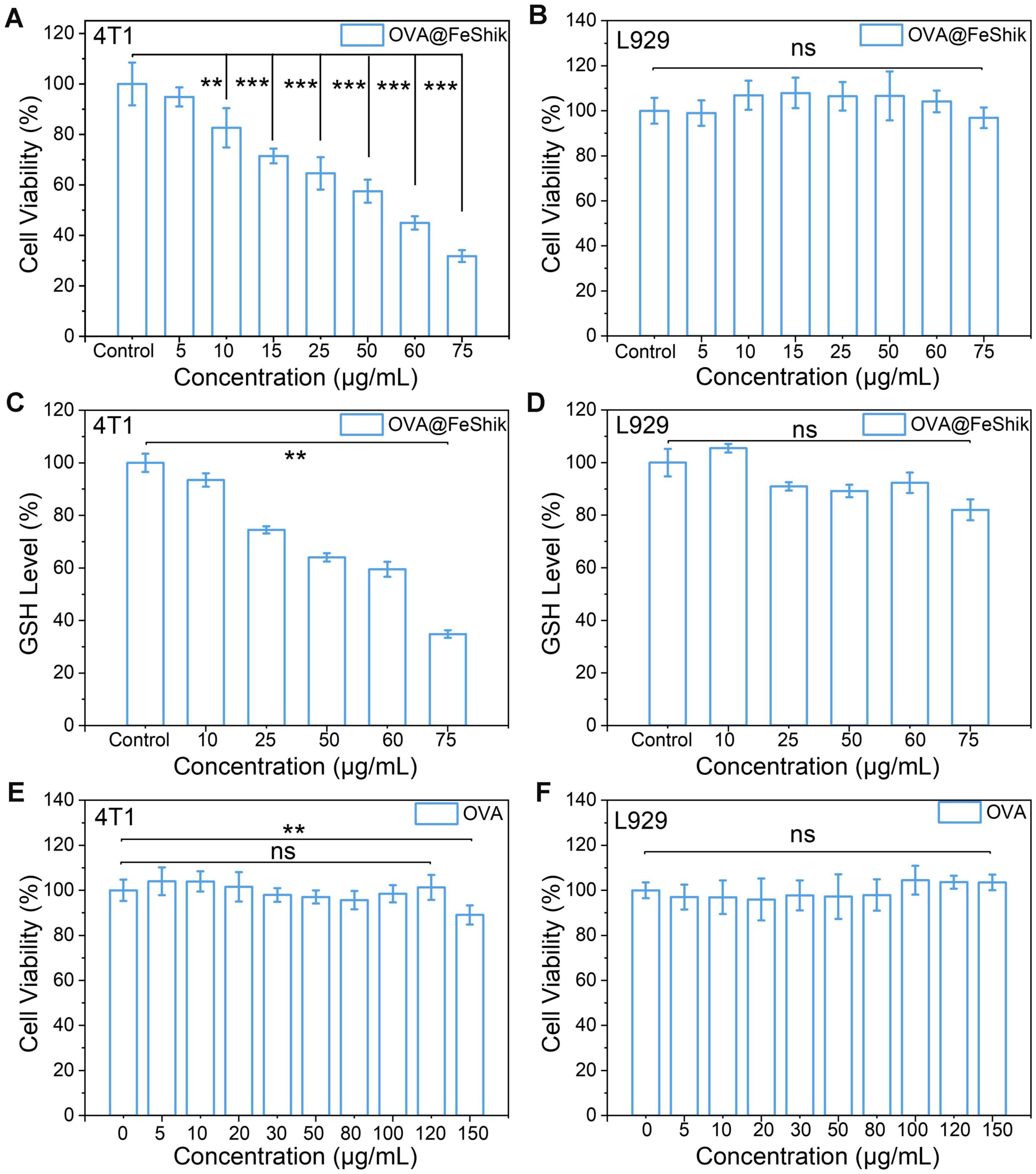

Figure S7. CLSM images (A), corresponding fluorescence intensity (B) (n = 3) and Pearson's R values (C-D) of 4T1 cells after incubation with ^{FITC}OVA and ^{FITC}OVA@FeShik (at an equivalent dosage of 60 μg/mL OVA@FeShik) for 6 h. Data are shown as mean ± SD; n represents the number of biologically independent samples.

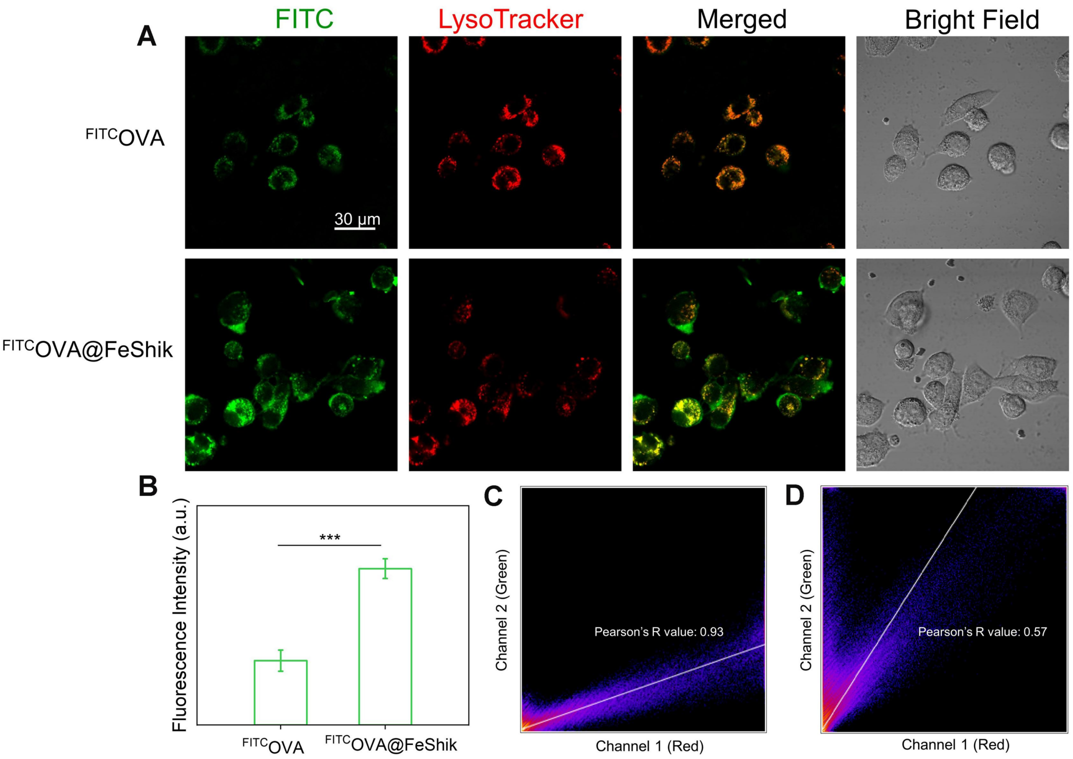

Figure S8. (A) CLSM examination of the level of ROS in 4T1 cells after treating with OVA, FeShik, and OVA@FeShik (at an equivalent dosage of 60 μg/mL OVA@FeShik). (B) The intensity of ROS fluorescence in 4T1 cells was analyzed by flow cytometry after treating with FeShik and OVA@FeShik.

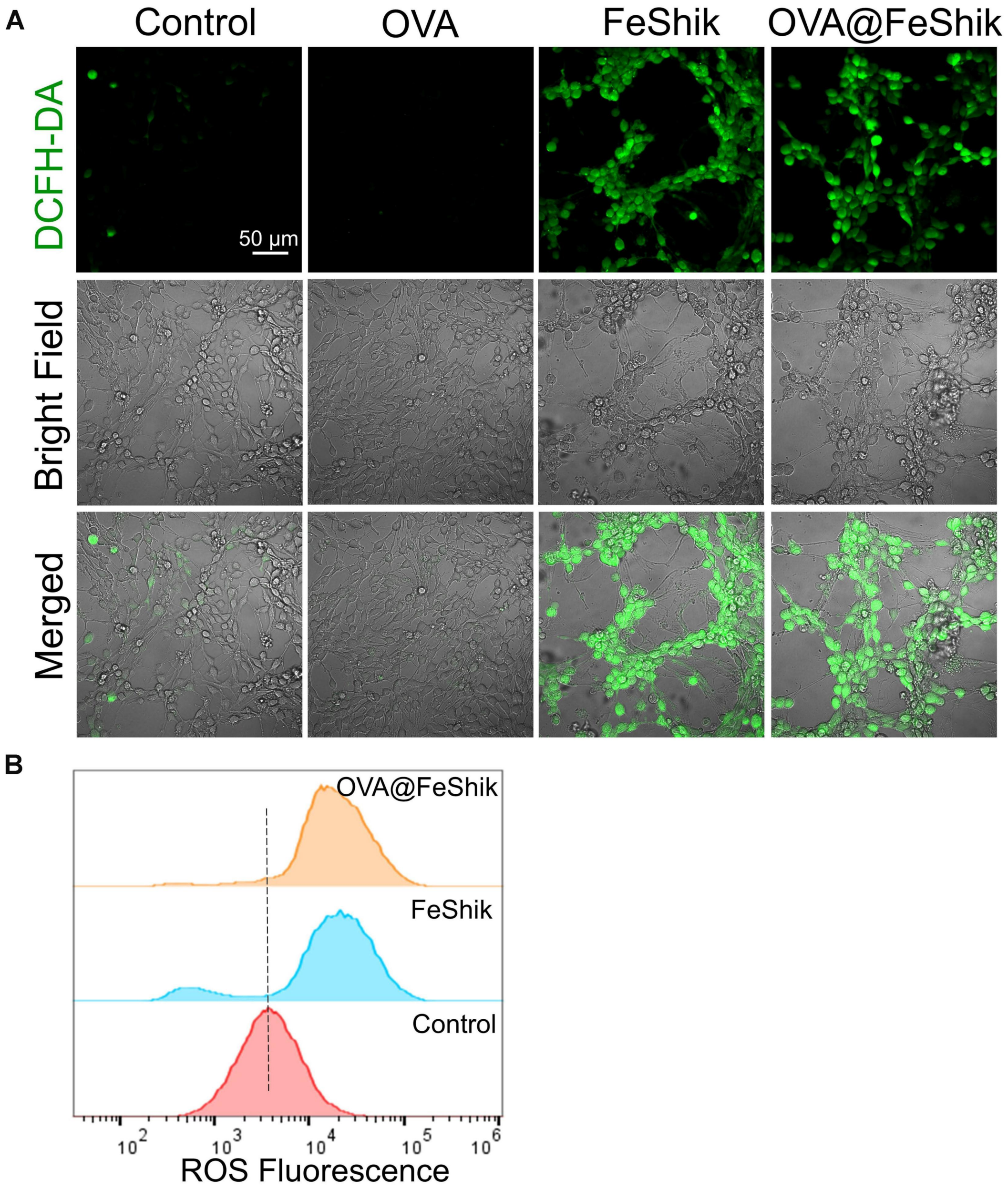

Figure S9. Cell viability of OVA@FeShik (60 µg/mL) treated 4T1 cells after the addition of Fer-1, Nec-1, and APO (n = 5). Data are shown as mean ± SD; n represents the number of biologically independent samples. *p < 0.05, **p < 0.01, and ***p < 0.001.

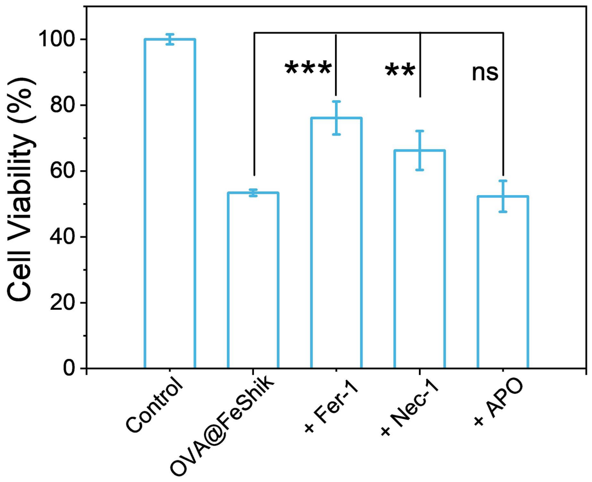

Figure S10. CLSM examination of Fe^{2+} level in 4T1 cells after treating with OVA, FeShik, and OVA@FeShik (at an equivalent dosage of 60 μg/mL OVA@FeShik).

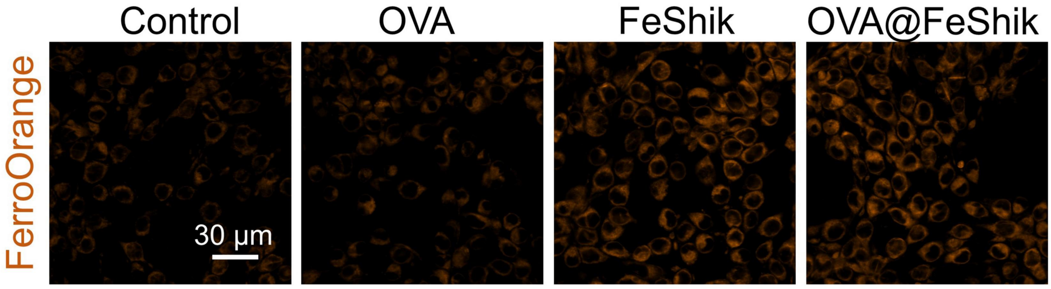

Figure S11. GSH content of 4T1 cells after treating with OVA, FeShik, and OVA@FeShik (at an equivalent dosage of 60 μg/mL OVA@FeShik) ($n = 3$). Data are shown as mean ± SD; n represents the number of biologically independent samples. *$p < 0.05$, **$p < 0.01$, and ***$p < 0.001$.

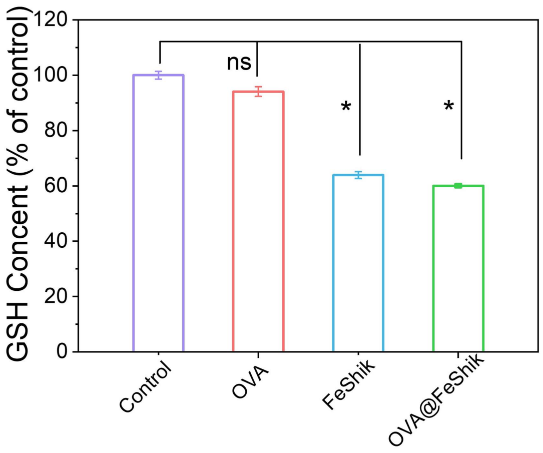

Figure S12. (A) CLSM examination of GPX4 expression in 4T1 cells after treating with OVA, FeShik, and OVA@FeShik (at an equivalent dosage of 60 µg/mL OVA@FeShik). (B) Quantification of GPX4 expression by ELISA assay kit after different treatments (n = 3). (C) Measurement of GPX4 expression by western blot in 4T1 cells after treating with OVA, FeShik, and OVA@FeShik (at an equivalent dosage of 60 µg/mL OVA@FeShik). Data are shown as mean ± SD; n represents the number of biologically independent samples. *p < 0.05, **p < 0.01, and ***p < 0.001.

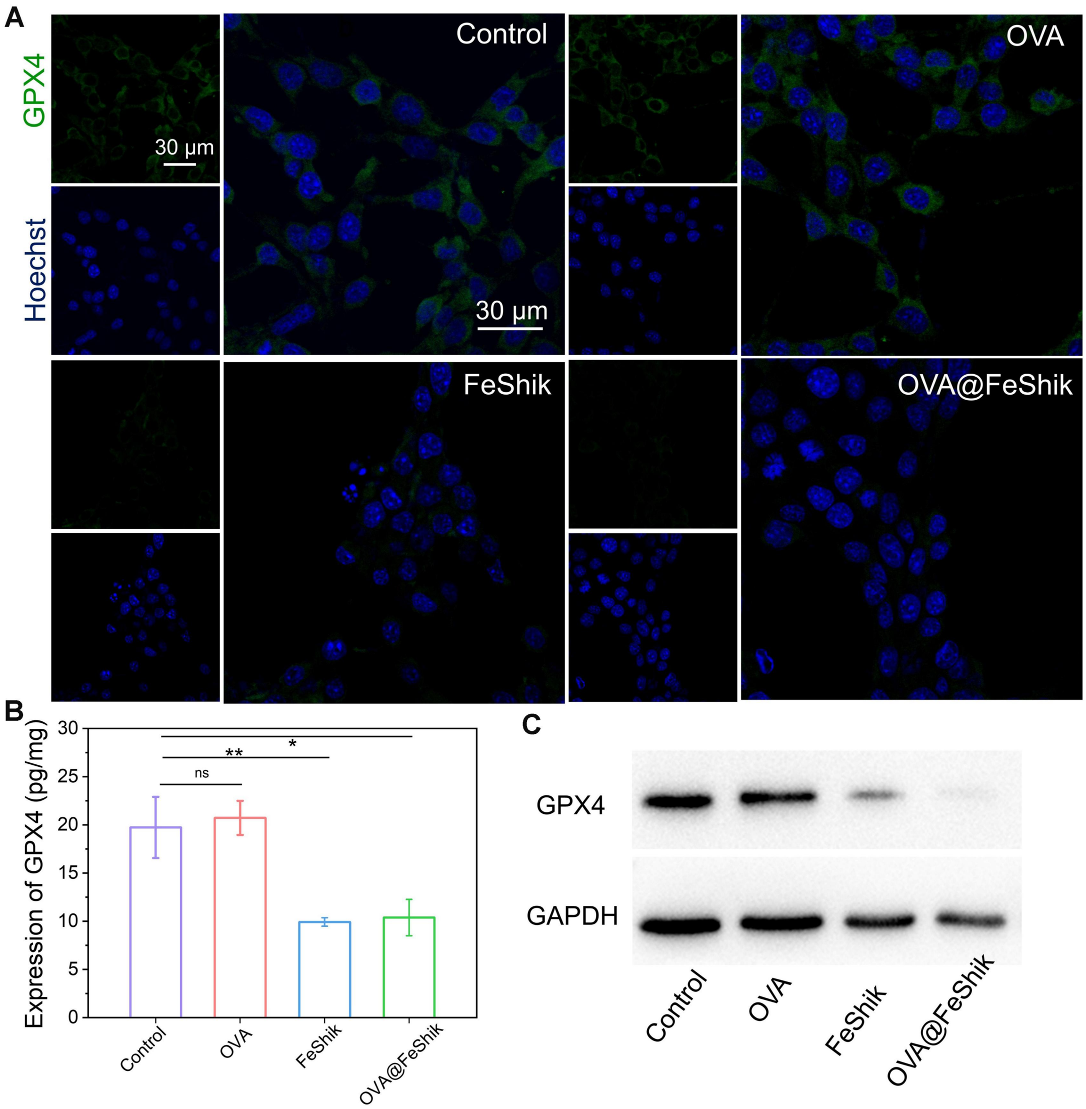

Figure S13. CLSM examination of LPO accumulation in 4T1 cells after treating with OVA, FeShik, and OVA@FeShik (at an equivalent dosage of 60 μg/mL OVA@FeShik).

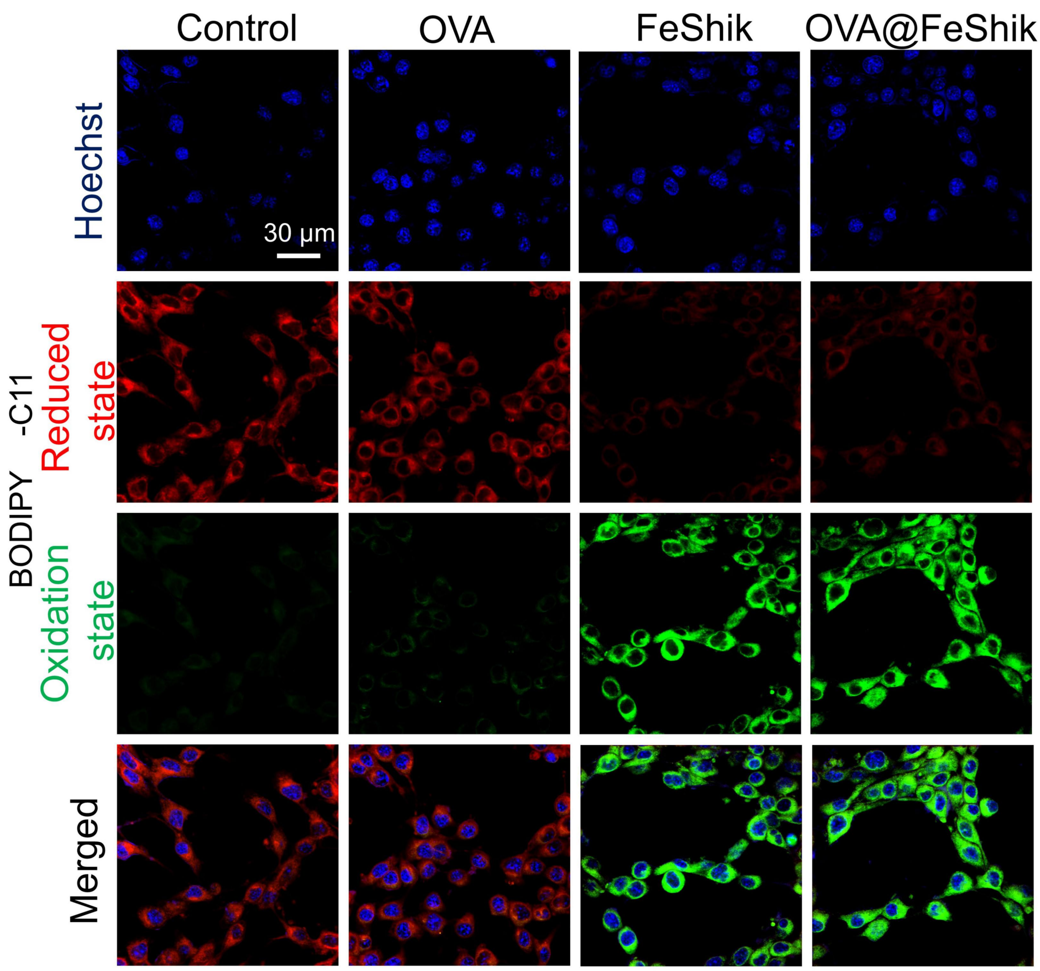

Figure S14. CLSM examination of RIP1 expression in 4T1 cells after treating with OVA, FeShik, and OVA@FeShik (at an equivalent dosage of 60 μg/mL OVA@FeShik).

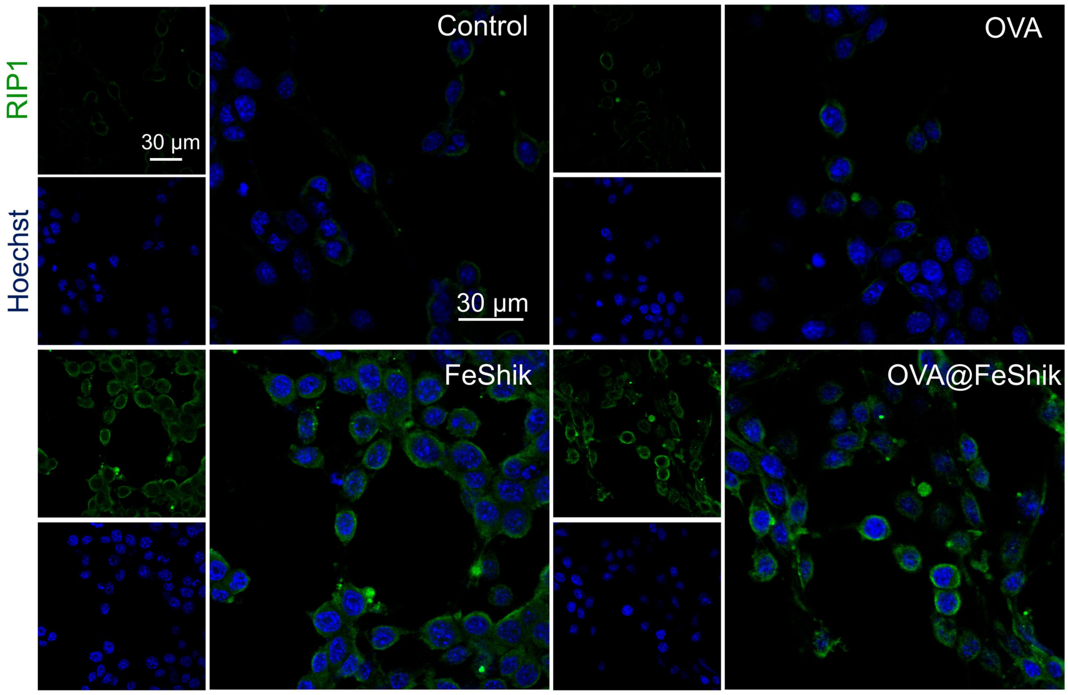

Figure S15. CLSM examination of RIP3 expression in 4T1 cells after treating with OVA, FeShik, and OVA@FeShik (at an equivalent dosage of 60 μg/mL OVA@FeShik).

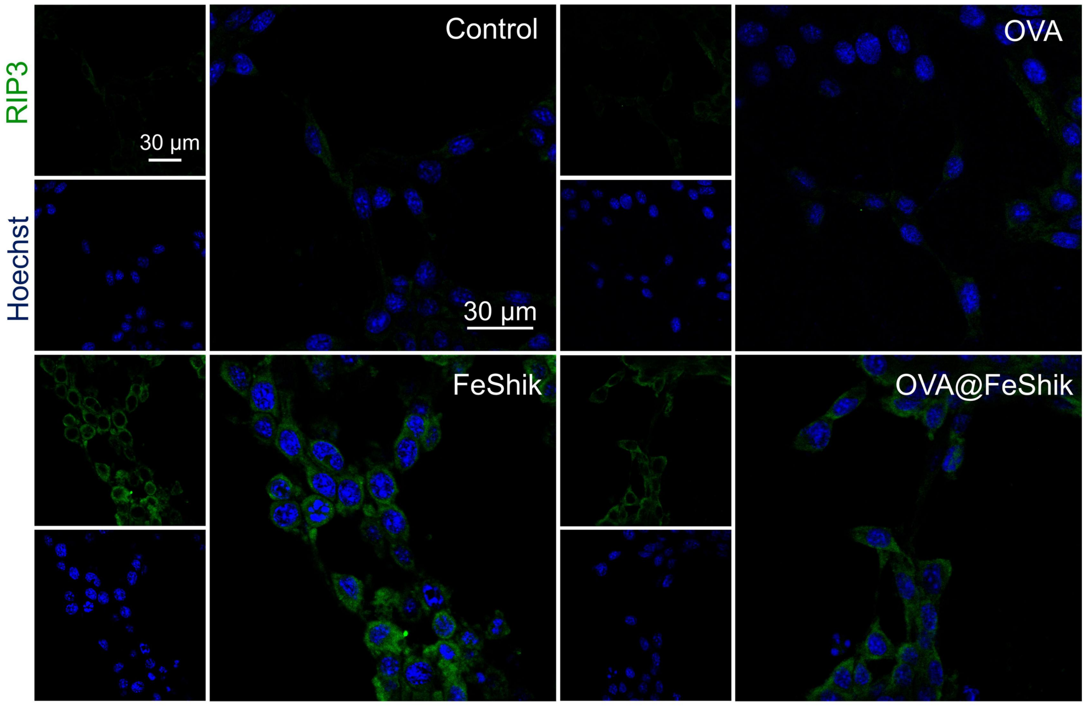

Figure S16. Expression of RIP1 (A) and RIP3 (B) analyzed by ELISA assay kit after treating with OVA, FeShik, and OVA@FeShik (at an equivalent dosage of 60 μg/mL OVA@FeShik) ($n = 3$). Data are shown as mean ± SD; n represents the number of biologically independent samples. *$p < 0.05$, **$p < 0.01$, and ***$p < 0.001$.

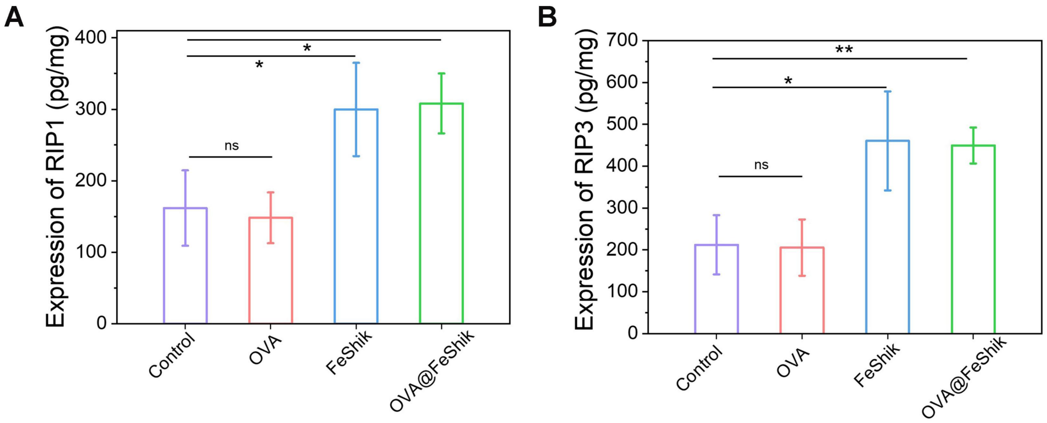

Figure S17. Measurement of RIP1 (A) and RIP3 (B) expression by western blot in 4T1 cells after treating with OVA, FeShik, and OVA@FeShik (at an equivalent dosage of 60 μg/mL OVA@FeShik).

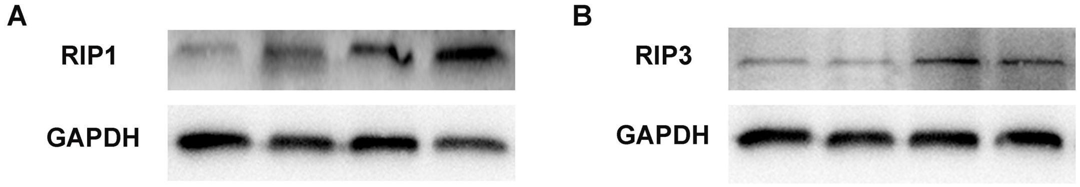

Figure S18. Annexin V-FITC/PI assay of 4T1 cells treated with 0 μg/mL (A), 5 μg/mL (B), 10 μg/mL (C), 15 μg/mL (D), 20 μg/mL (E) and 25 μg/mL (F) of OVA@FeShik.

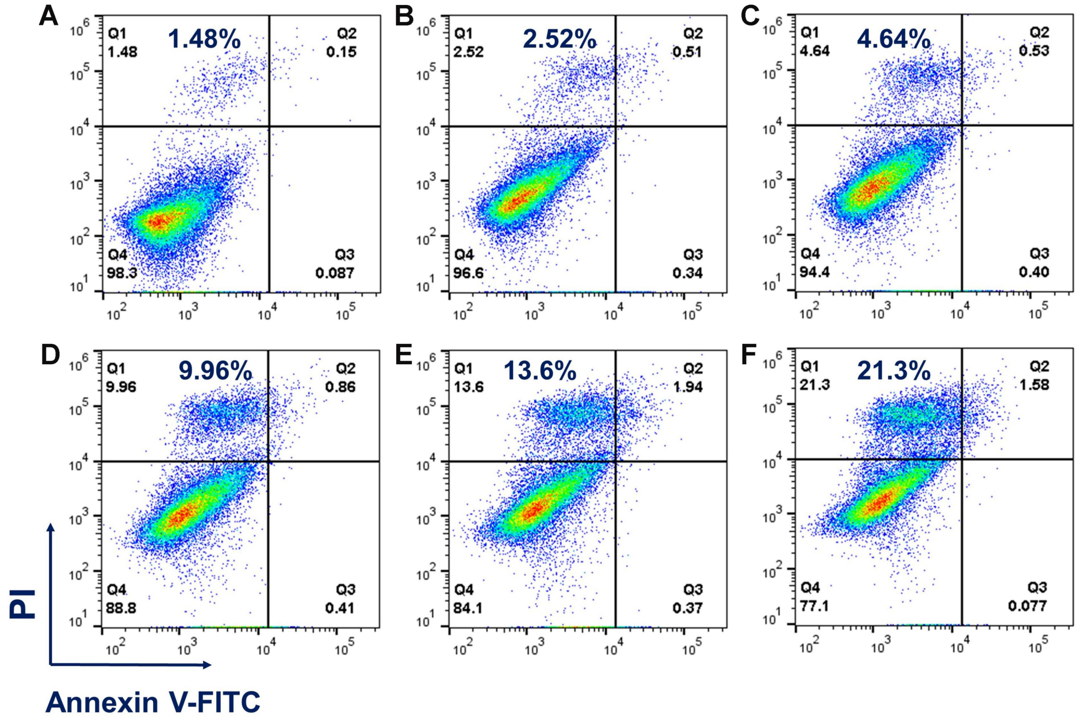

Figure S19. Measurement of CRT and HMGB1 expression by western blot in 4T1 cells after treating with OVA, FeShik, and OVA@FeShik (at an equivalent dosage of 60 μg/mL OVA@FeShik).

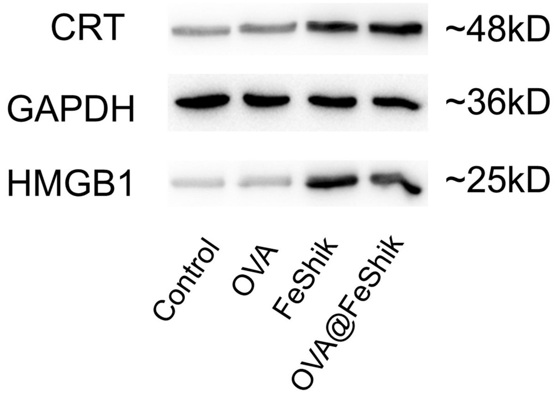

Figure S20. CLSM images of BMDCs (A) and BMDMs (B) after treating with 4T1 cells and ^{FITC}OVA@FeShik in transwell system.

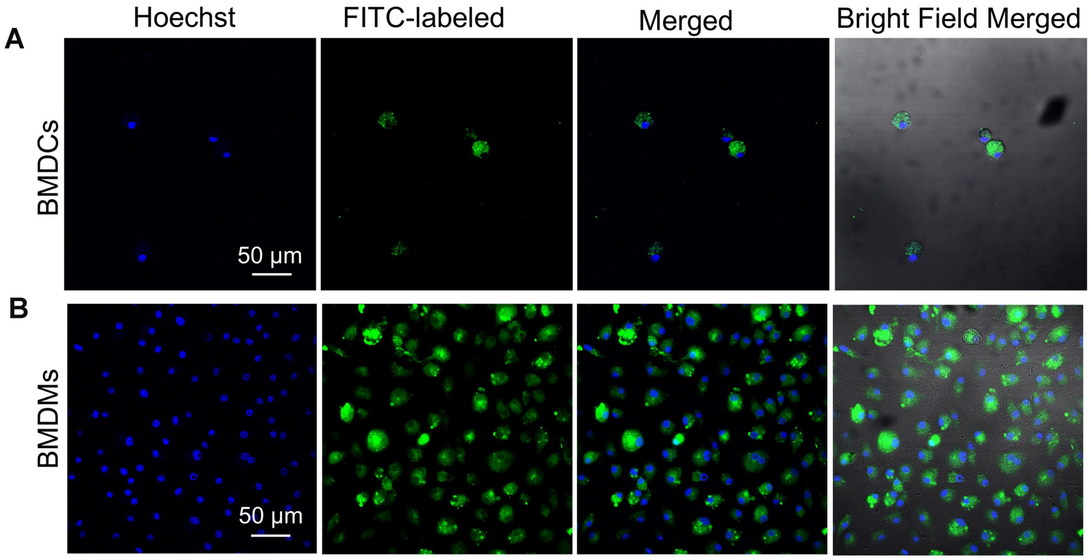

Figure S21. Blood circulation profiles of ^{IR780}OVA (A), ^{IR780}FeShik (B), and ^{IR780}OVA@FeShik (C) in BALB/c mice by recording the IR780 fluorescence intensity of blood samples at different time points (n = 3). Data are shown as mean ± SD; n represents the number of biologically independent samples.

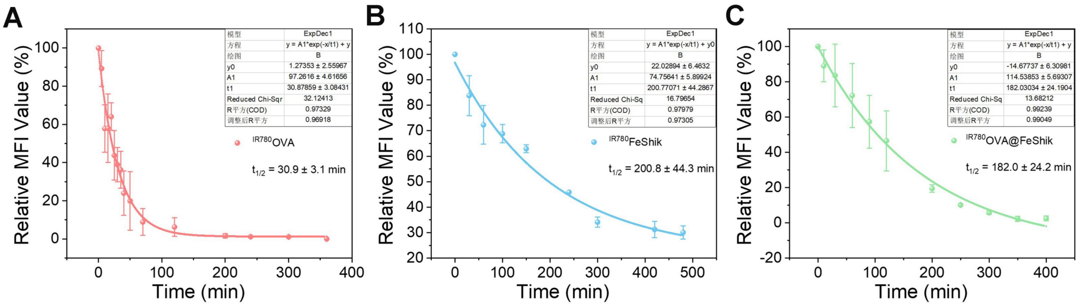

Figure S22. Quantification analysis of ^{IR780}OVA (A), ^{IR780}FeShik (B), and ^{IR780}OVA@FeShik (C) in tumor and major organs by testing the corresponding fluorescence intensity after intravenous injection for 96 h (n = 3). Data are shown as mean ± SD; n represents the number of biologically independent samples.

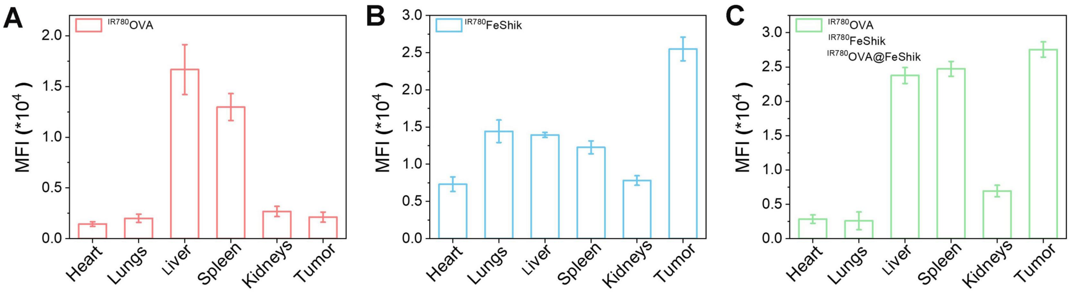

Figure S23. Photographs of tumors in each group after different treatments.

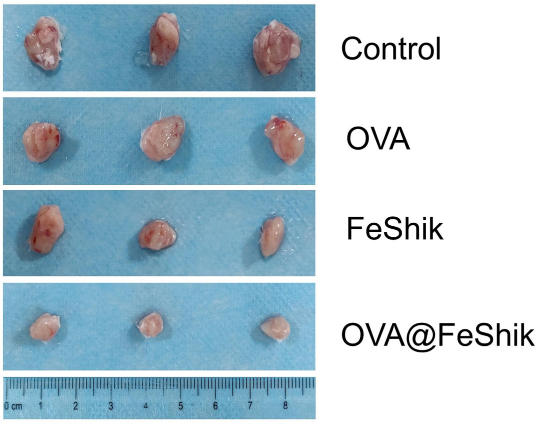

Figure S24. Quantitative analysis of the M2-phenotype macrophages ($F4/80^{+}CD206^{+}CD86^{-}$) (A) and M1-phenotype macrophages ($F4/80^{+}CD206^{-}CD86^{+}$) (B) by flow cytometry assay in tumors after different treatments (n = 3). Data are shown as mean ± SD; n represents the number of biologically independent samples. *p < 0.05, **p < 0.01, and ***p < 0.001.

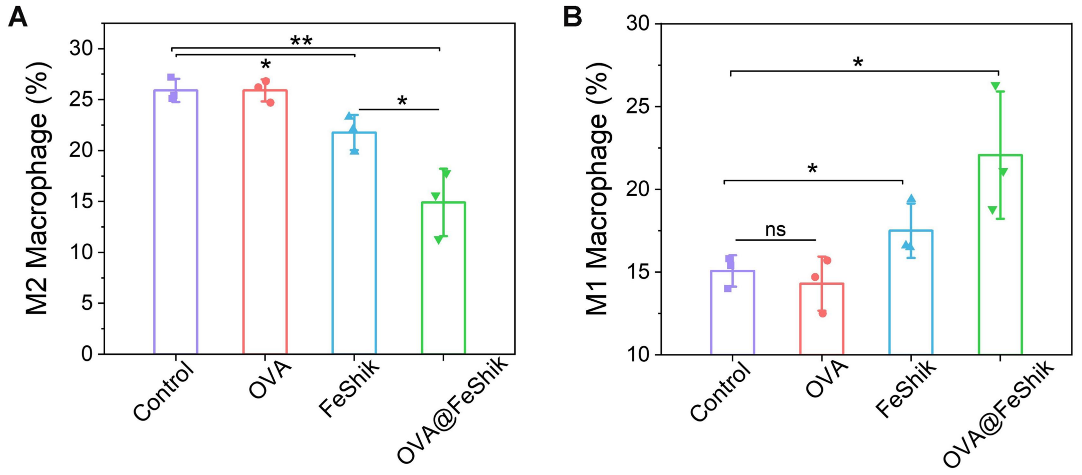

Figure S25. $CD4^+$ T cells staining images for tumors after various treatments.

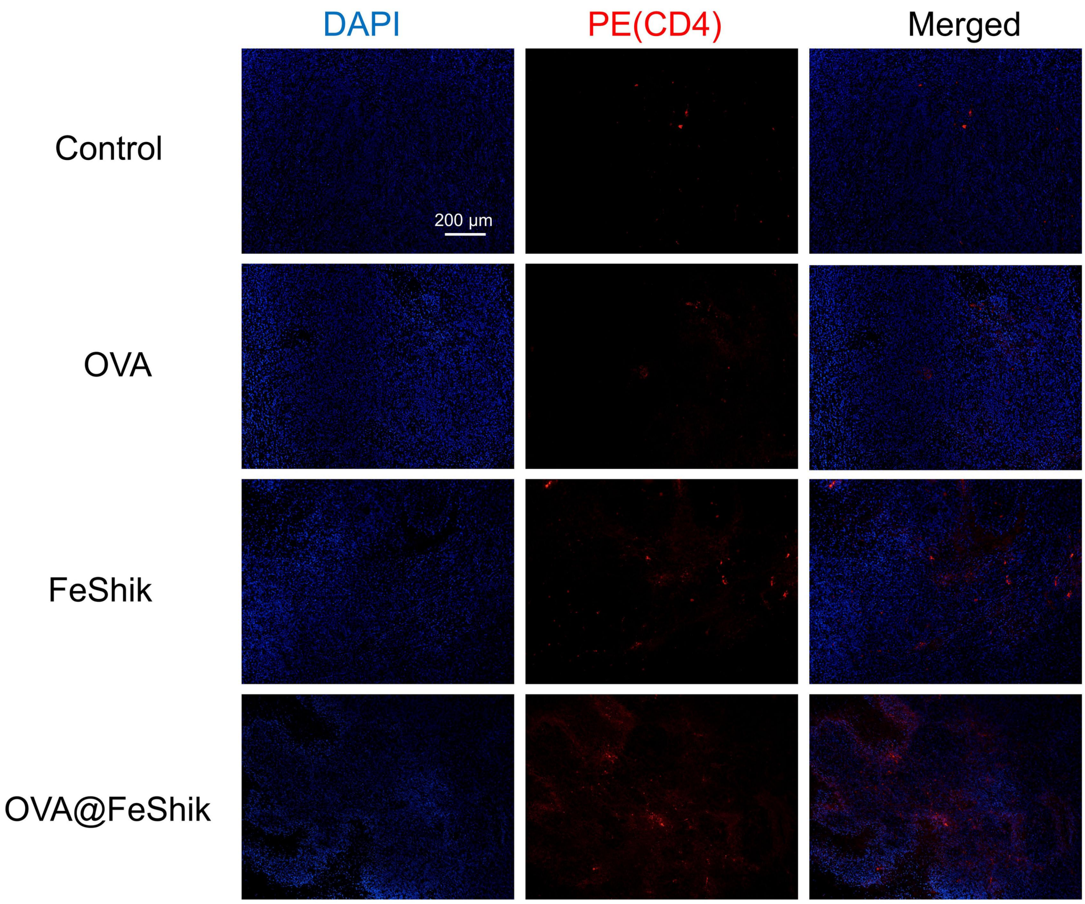

Figure S26. $CD8^+$ T cells staining images for tumors after various treatments.

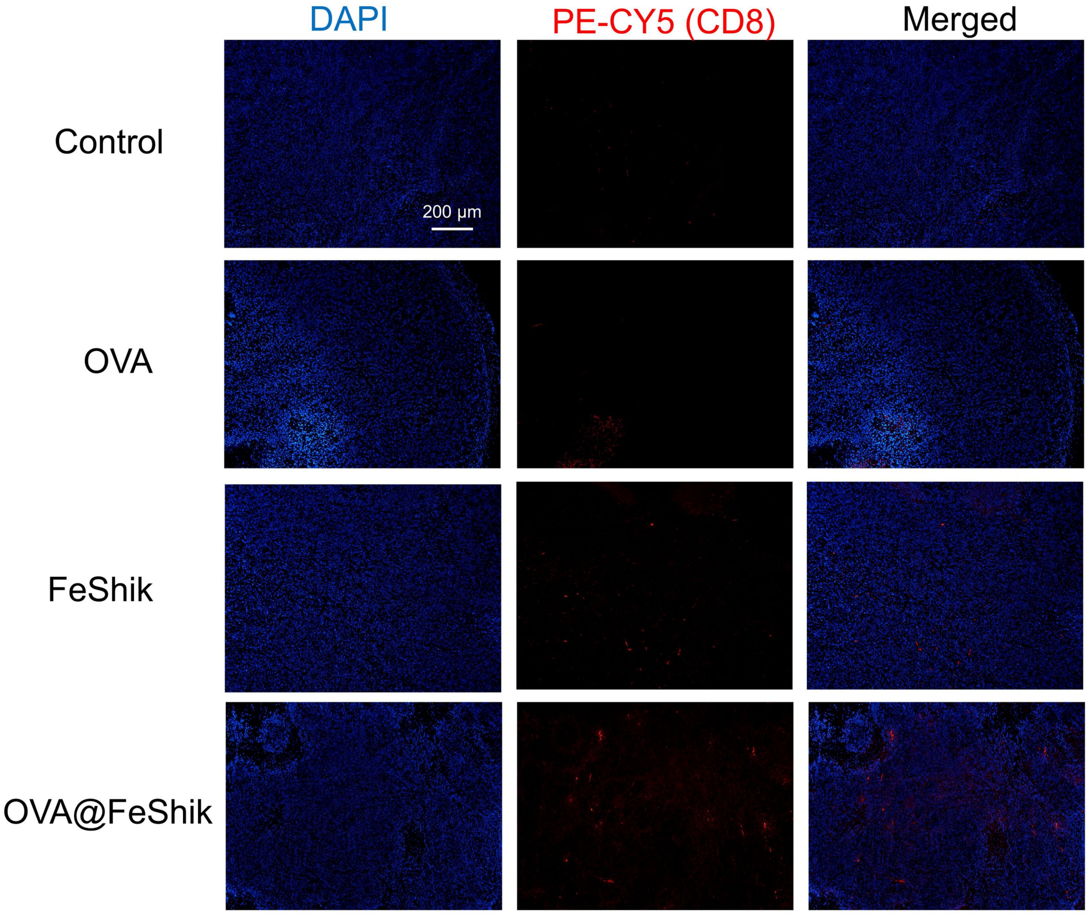

Table S1. The protein composition of extracted TF is characterized by UHPLC-MS/MS analyses. A total of 2451 proteins are detected, and the information of the top eight proteins are given.

	**Protein**	**Description**	**Content (%)**	**Mass (kDa)**
**1**	P16045	Galectin-1	4.69	14.866
**2**	Q5FW97	phosphopyruvate hydratase	2.68	47.14
**3**	Q4KL81	Actin, gamma, cytoplasmic 1	2.56	41.792
**4**	P62983	Ubiquitin-40S ribosomal protein S27a	1.92	17.951
**5**	Q5FWJ3	Vimentin	1.56	53.687
**6**	P05213	Tubulin alpha-1B chain	1.51	50.151
**7**	Q3U6E4	Prothymosin alpha	1.38	12.325
**8**	Q564E2	L-lactate dehydrogenase	1.36	36.498

Figure S27. TEM image (A), size distribution (B), and hydrated diameter (C) of TF@FeShik. (D) UV-vis absorption spectra of TF, FeShik, and TF@FeShik.

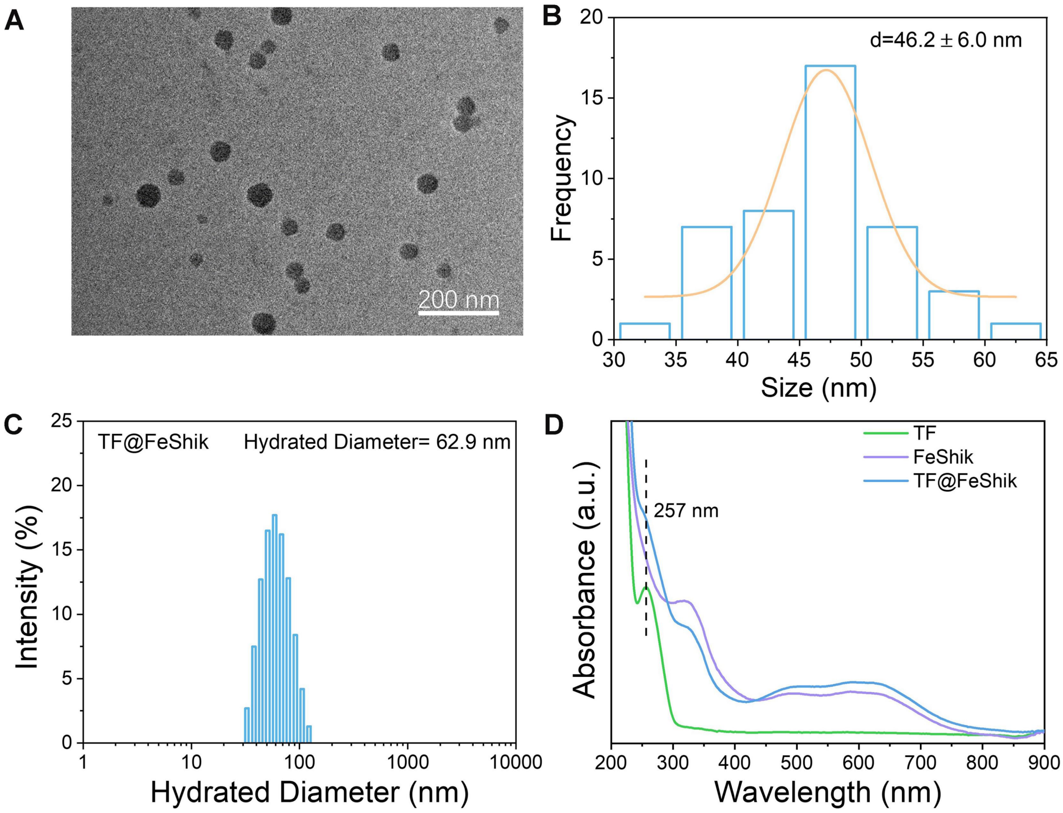

Figure S28. SDS-PAGE protein analysis by Coomassie Brilliant Blue staining of TF, TF@FeShik, and FeShik.

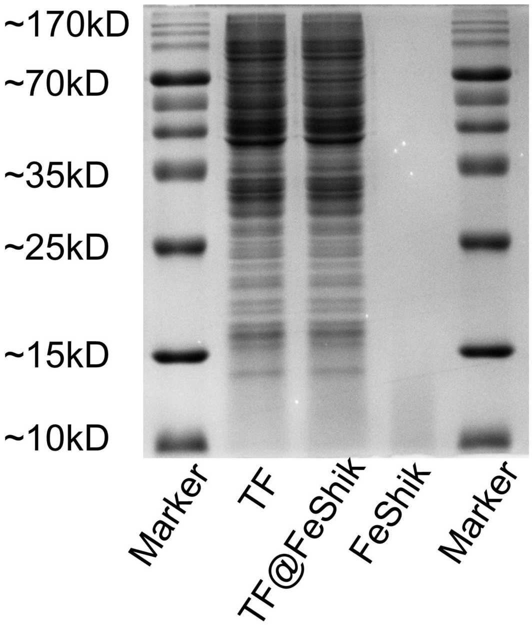

Figure S29. Standard absorption curve of TF according to the concentration and UV-vis absorbance at 257 nm.

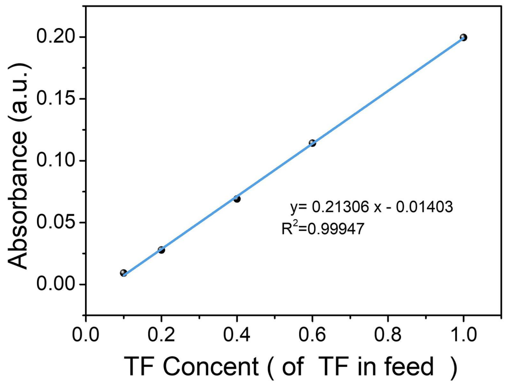

Figure S30. The release curve of Fe^{2+} (A), Shikonin (B), and TF (C) from TF@FeShik nanovaccines after incubation with or without 10 mM GSH (n = 3). Data are shown as mean ± SD; n represents the number of biologically independent samples.

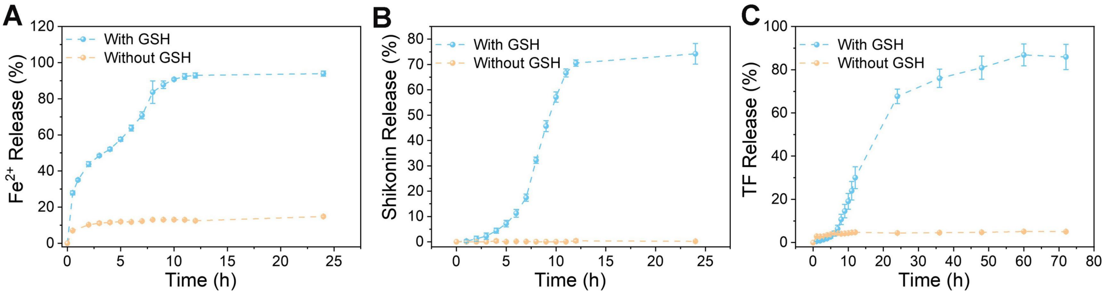

Figure S31. H&E staining images of distant tumor tissue slices from mice after different treatments.

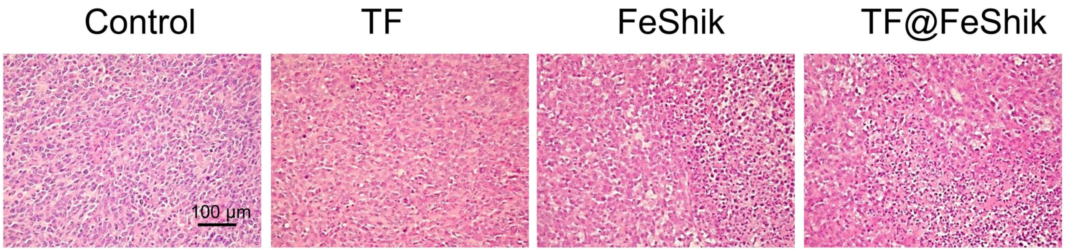

Figure S32. Immunofluorescence staining images (GPX4, RIP1, RIP3, and HMGB1) of primary tumor tissue slices after different treatments.

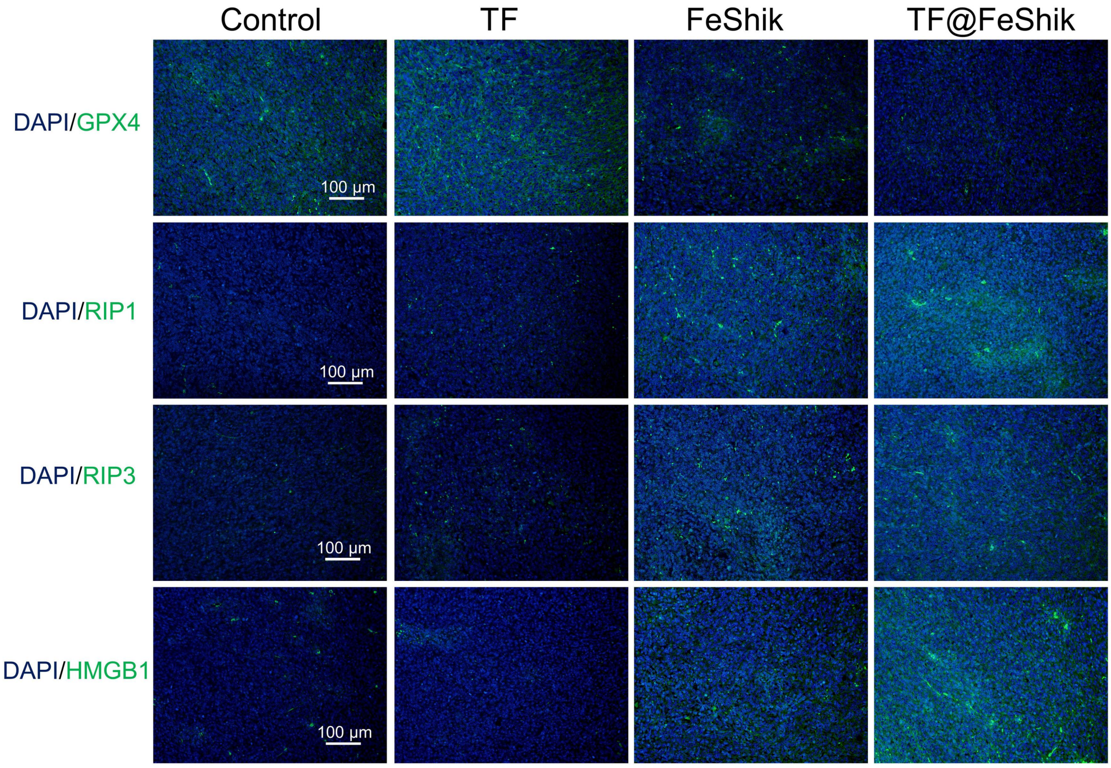

Figure S33. Photographs of primary tumors (A) and distant tumors (B) in each group after different treatments.

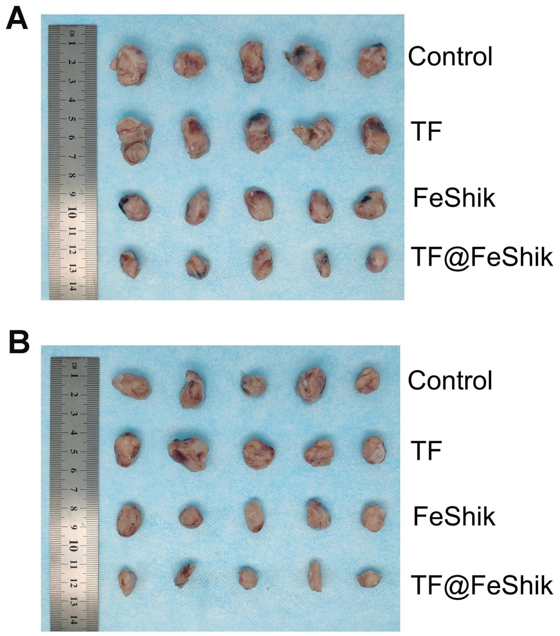

Figure S34. Individual body weight curves of mice in each group after various treatments (n = 15).

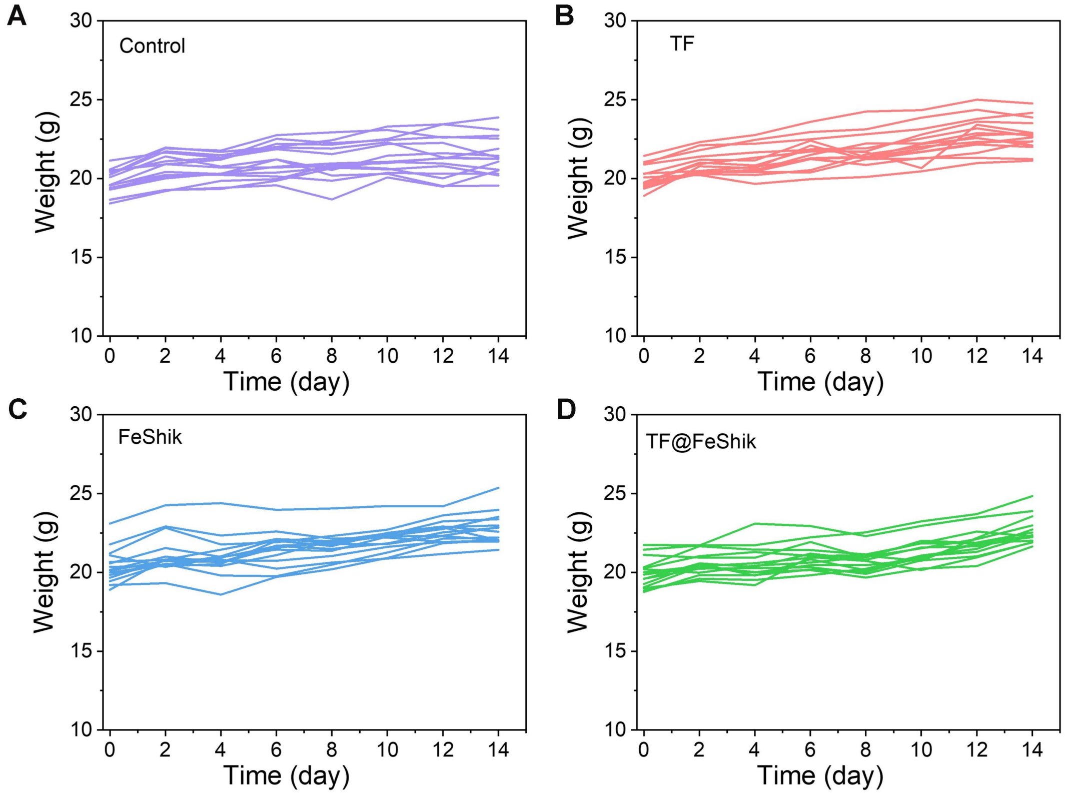

Figure S35. Flow cytometric analysis of DC maturation ($CD11c^{+}CD80^{+}CD86^{+}$) in spleens after different treatments.

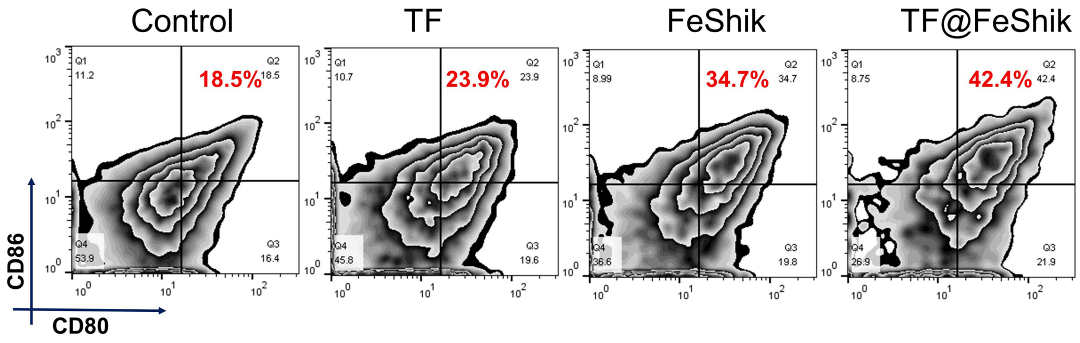

Figure S36. Flow cytometric analysis of T helper cells ($CD3^+CD4^+$) (A) and cytotoxic T lymphocytes ($CD3^+CD8^+$) (B) in primary tumors after different treatments.

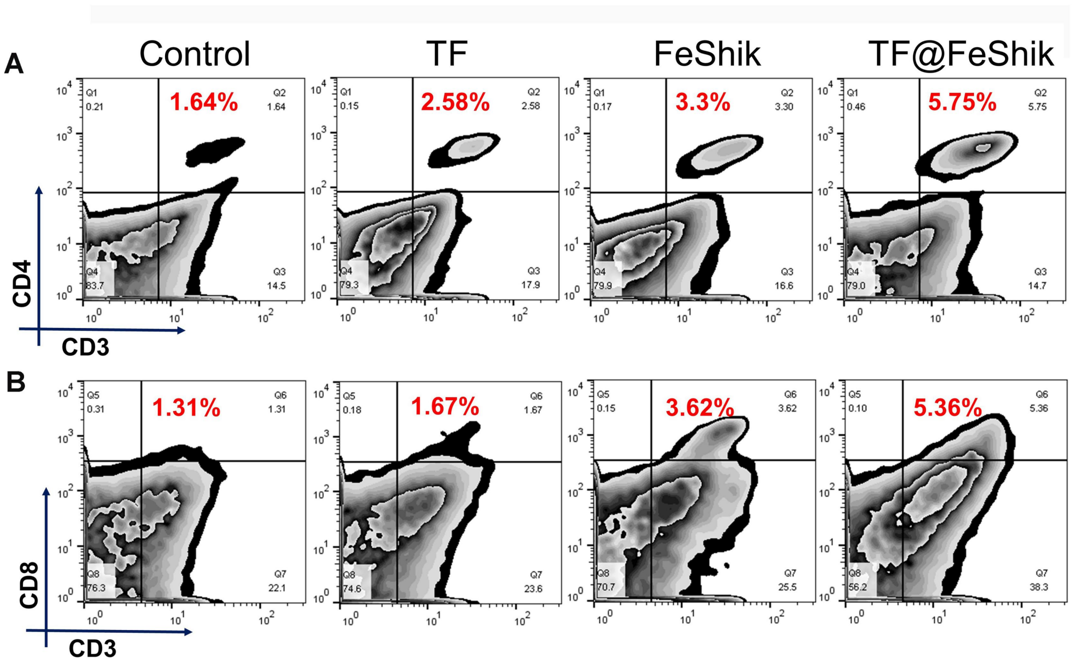

Figure S37. Flow cytometric analysis of T helper cells ($CD3^+CD4^+$) (A) and cytotoxic T lymphocytes ($CD3^+CD8^+$) (B) in distant tumors after different treatments.

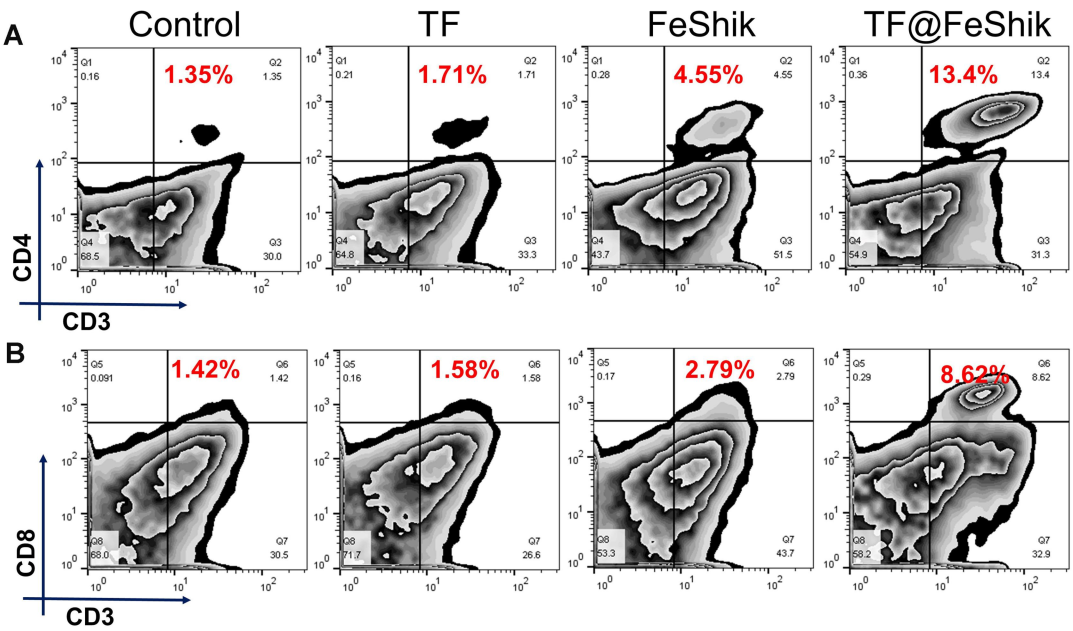

Figure S38. Immunofluorescence staining images (CD4 and CD8) of primary tumor tissue slices from mice sacrificed at 14th day after different treatments.

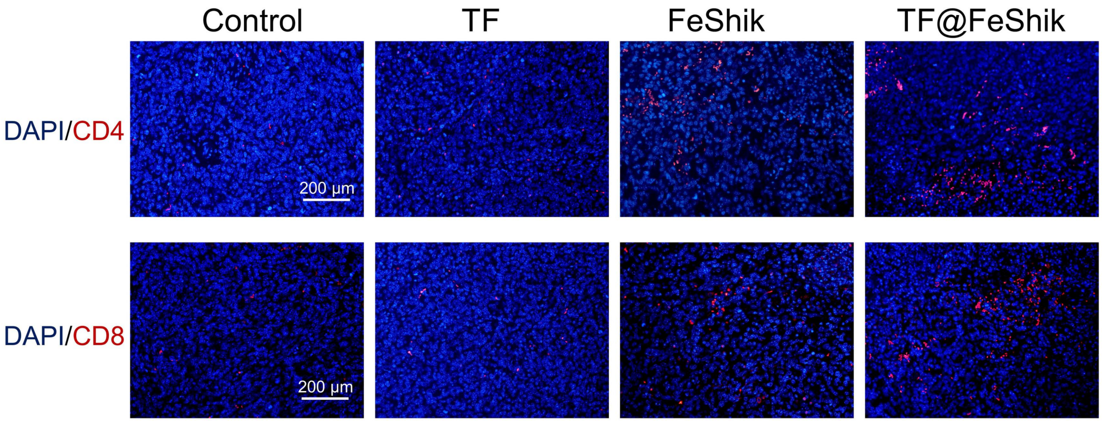

Figure S39. Flow cytometric analysis (A) and the corresponding quantitative analysis of M2-phenotype macrophages (F4/80$^+$CD206$^+$CD86$^-$) (B) and M1-phenotype macrophages (F4/80$^+$CD206$^-$CD86$^+$) (C) in primary tumors after different treatments (n = 3). Data are shown as mean ± SD; n represents the number of biologically independent samples. *$p < 0.05$, **$p < 0.01$, and ***$p < 0.001$.

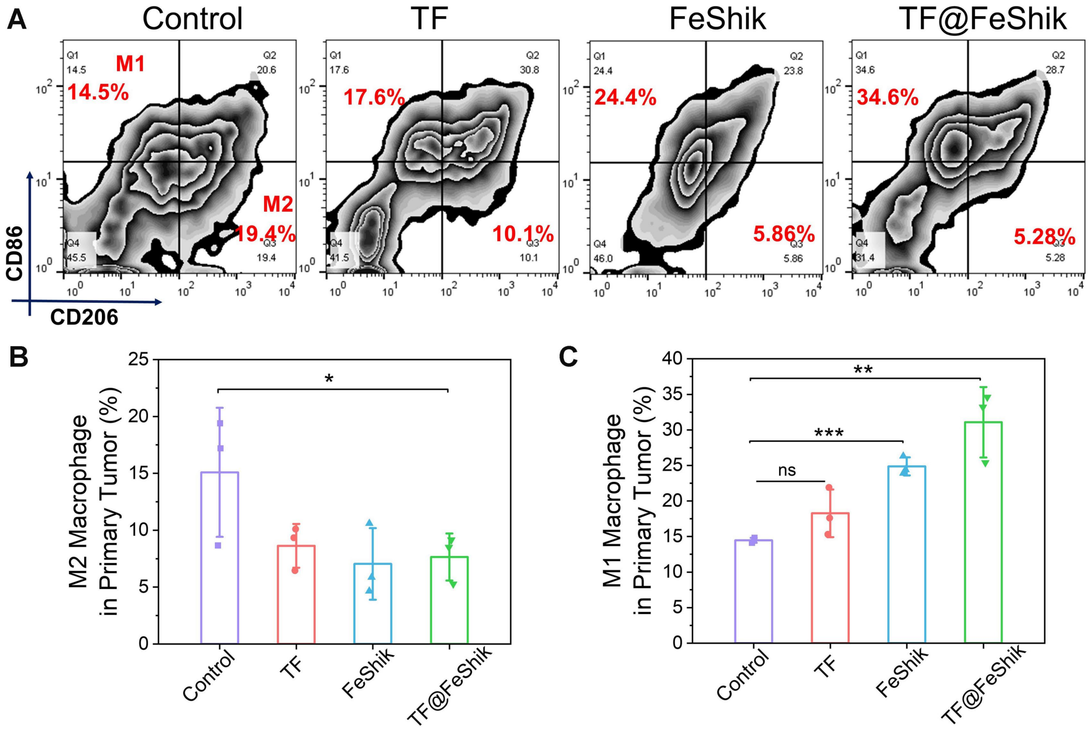

Figure S40. Flow cytometric analysis (A) and the corresponding quantitative analysis of M2-phenotype macrophages ($F4/80^{+}CD206^{+}CD86^{-}$) (B) and M1-phenotype macrophages ($F4/80^{+}CD206^{-}CD86^{+}$) (C) in distant tumors after different treatments(n = 3). Data are shown as mean ± SD; n represents the number of biologically independent samples. *$p < 0.05$, **$p < 0.01$, and ***$p < 0.001$.

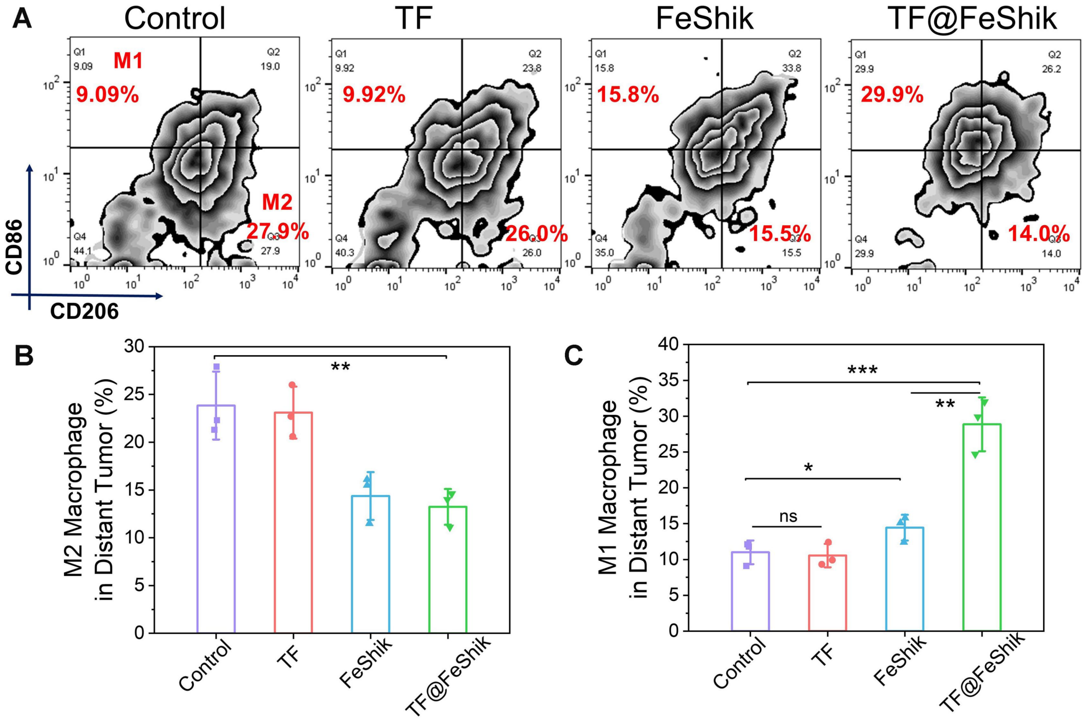

Figure S41. (A) In vitro T_1-weighted MRI signals of TF@FeShik with and without GSH. (B) MRI images of 4T1 tumor-bearing BALB/c mice without or with TF@FeShik treatment.

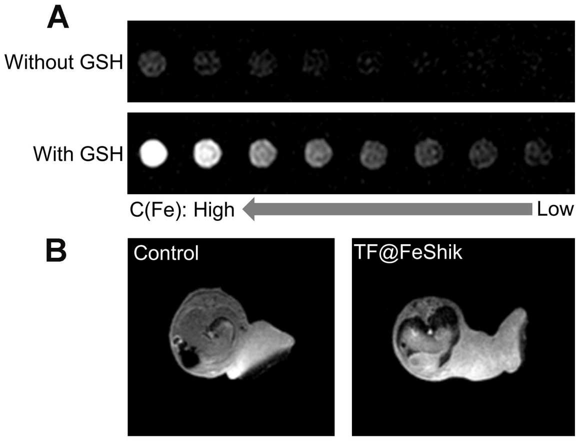

Figure S42. H&E stained splanchnic slices of heart, lungs, liver, spleen, and kidneys of mice after different treatments.

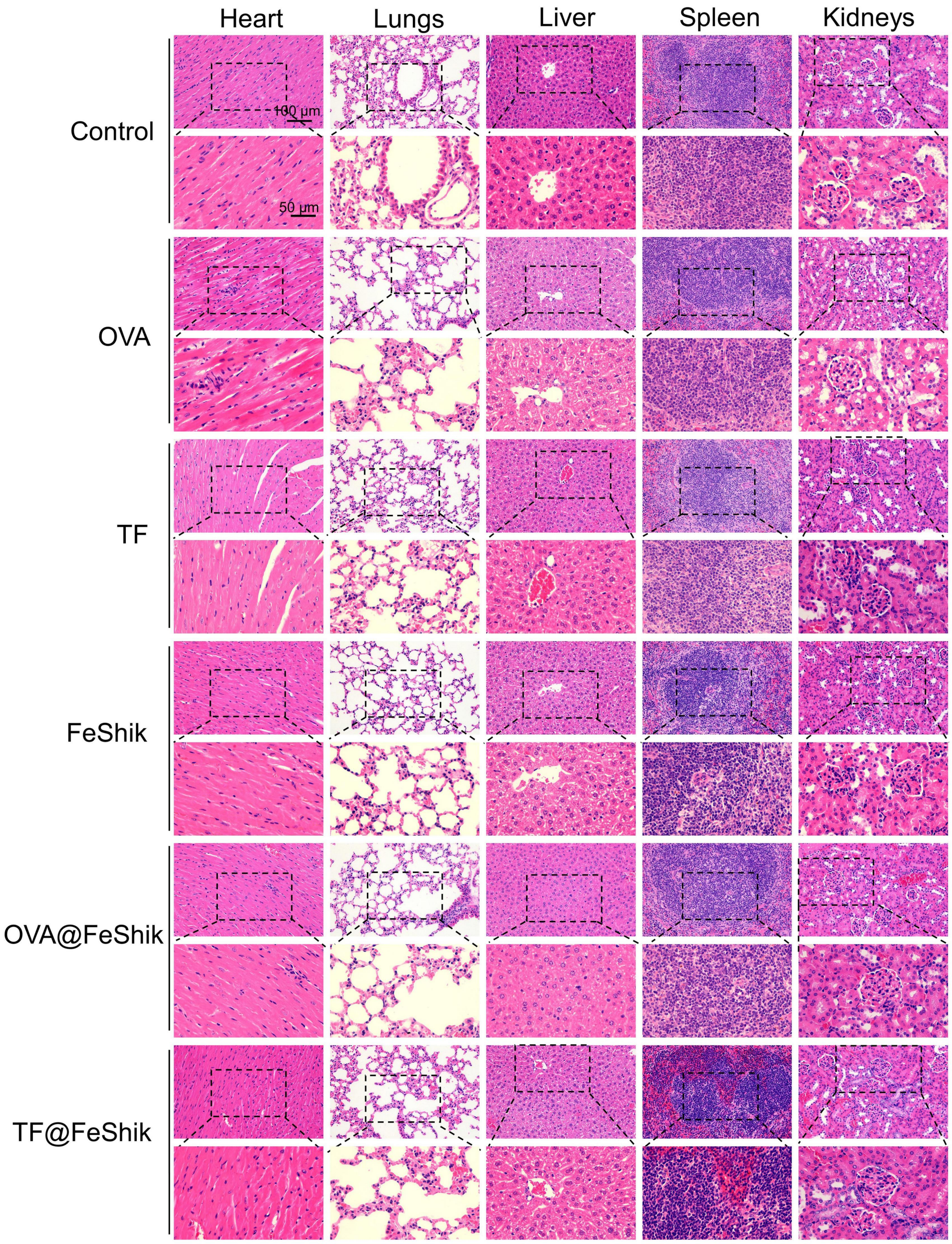

Figure S43. Main indexes of liver and renal functions tests of mice in each group (n = 3). (A) Total protein (TP), (B) albumin (ALB), (C) globulins (GLO), (D) albumin-globulin ratio (A/G), (E) alanine aminotransferase (ALT), (F) aspartate transaminase (AST), (G) alkaline phosphatase, (H) total bile acid (TBA), (I) cholinesterase, (J) uric acid (UA), (K) creatinine, (L) Phosphorus, (M) Magnesium, and (N) total calcium (tCa). Data are shown as mean ± SD; n represents the number of biologically independent samples.

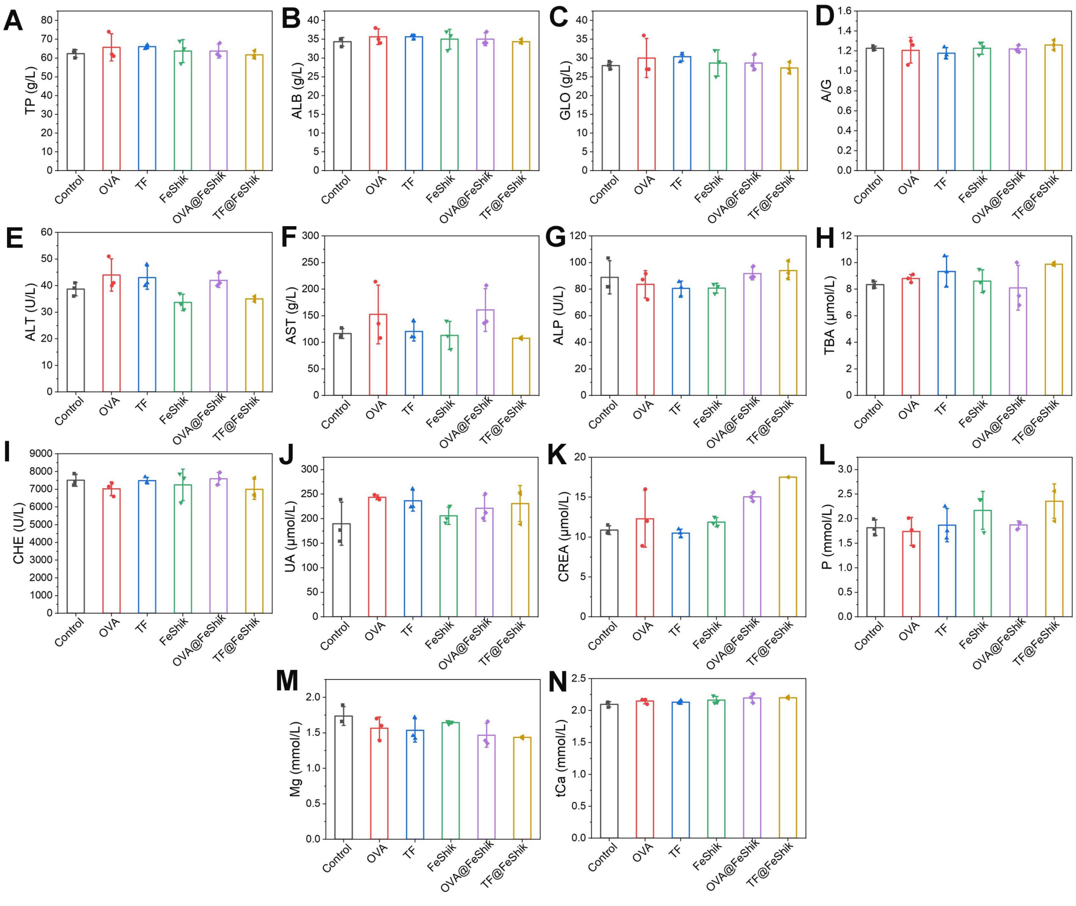

Supplement: Supplementary file 1 — Supplementary figures. [file thnov13p5266s1.pdf]
